# Adoptive NK Cell Transfer as a Treatment in Colorectal Cancer Patients: Analyses of Tumour Cell Determinants Correlating With Efficacy *In Vitro* and *In Vivo*

**DOI:** 10.3389/fimmu.2022.890836

**Published:** 2022-06-07

**Authors:** Pilar M. Lanuza, M. Henar Alonso, Sandra Hidalgo, Iratxe Uranga-Murillo, Sandra García-Mulero, Raquel Arnau, Cristina Santos, Xavier Sanjuan, Llipsy Santiago, Laura Comas, Sergio Redrado, Roberto Pazo-Cid, M. Jose Agustin-Ferrández, Paula Jaime-Sánchez, Cecilia Pesini, Eva M. Gálvez, Ariel Ramírez-Labrada, Maykel Arias, Rebeca Sanz-Pamplona, Julián Pardo

**Affiliations:** ^1^ Aragón Health Research Institute (IIS Aragón), Biomedical Research Centre of Aragón (CIBA), Zaragoza, Spain; ^2^ Unit of Biomarkers and Susceptibility, Oncology Data Analytics Program (ODAP), Catalan Institute of Oncology (ICO), Oncobell Program, Bellvitge Biomedical Research Institute (IDIBELL) and CIBERESP, Hospitalet de Llobregat, Barcelona, Spain; ^3^ Department of Microbiology, Radiology, Pediatry and Public Health, University of Zaragoza, Zaragoza, Spain; ^4^ CIBER de Enfermedades Infecciosas, Instituto de Salud Carlos III, Madrid, Spain; ^5^ Department of Clinical Sciences, Faculty of Medicine, University of Barcelona, Barcelona, Spain; ^6^ Department of Medical Oncology, Catalan Institute of Oncology (ICO), Oncobell Program, Bellvitge Biomedical Research Institute (IDIBELL)-CIBERONC, L’Hospitalet de Llobregat, Barcelona, Spain; ^7^ Department of Pathology, University Hospital Bellvitge (HUB-IDIBELL), L’Hospitalet de Llobregat, Barcelona, Spain; ^8^ Oncology and Pharmacology Units, HUMSICB-CSIC, Instituto de Carboquímica ICB-CSIC, Zaragoza, Spain; ^9^ Hospital Universitario Miguel Servet, Zaragoza, Spain; ^10^ Unidad de Nanotoxicología e Inmunotoxicología (UNATI), Aragón Health Research Institute (IIS Aragón), Biomedical Research Centre of Aragón (CIBA), Zaragoza, Spain; ^11^ ARAID Foundation, Aragon Health Research Institute (IIS Aragón), Zaragoza, Spain

**Keywords:** colorectal cancer, cancer biomarker, NK cell immunotherapy, tumour immune microenvironment, immune checkpoints, HLA-I

## Abstract

**Background:**

Colorectal cancer (CRC) is a heterogeneous disease with variable mutational profile and tumour microenvironment composition that influence tumour progression and response to treatment. While chemoresistant and poorly immunogenic CRC remains a challenge, the development of new strategies guided by biomarkers could help stratify and treat patients. Allogeneic NK cell transfer emerges as an alternative against chemoresistant and poorly immunogenic CRC.

**Methods:**

NK cell-related immunological markers were analysed by transcriptomics and immunohistochemistry in human CRC samples and correlated with tumour progression and overall survival. The anti-tumour ability of expanded allogeneic NK cells using a protocol combining cytokines and feeder cells was analysed *in vitro* and *in vivo* and correlated with CRC mutational status and the expression of ligands for immune checkpoint (IC) receptors regulating NK cell activity.

**Results:**

HLA-I downmodulation and NK cell infiltration correlated with better overall survival in patients with a low-stage (II) microsatellite instability-high (MSI-H) CRC, suggesting a role of HLA-I as a prognosis biomarker and a potential benefit of NK cell immunotherapy. Activated allogeneic NK cells were able to eliminate CRC cultures without PD-1 and TIM-3 restriction but were affected by HLA-I expression. *In vivo* experiments confirmed the efficacy of the therapy against both HLA^+^ and HLA^−^ CRC cell lines. Concomitant administration of pembrolizumab failed to improve tumour control.

**Conclusions:**

Our results reveal an immunological profile of CRC tumours in which immunogenicity (MSI-H) and immune evasion mechanisms (HLA downmodulation) favour NK cell immunosurveillance at early disease stages. Accordingly, we have shown that allogeneic NK cell therapy can target tumours expressing mutations conferring poor prognosis regardless of the expression of T cell-related inhibitory IC ligands. Overall, this study provides a rationale for a new potential basis for CRC stratification and NK cell-based therapy.

## Introduction

Immunotherapy has emerged as a new strategy in cancer treatment, with outstanding results after the introduction of immune checkpoint inhibitors (ICIs) (1). However, the global response rate to ICIs is relatively low, as their efficacy seems to be limited to immunogenic tumours, defined by their mutational burden and neoantigen expression, as well as the degree of immune infiltration (2). This limitation is exemplified by colorectal cancer (CRC), the third most common cancer and the second leading cause of cancer-related deaths. Only about 10%–20% of CRC tumours that present microsatellite instability (MSI) have good response rates (3).

T cells represent the main target population for ICIs. Therefore, sensitivity to ICIs has been associated with HLA-I and HLA-II expression on tumours and antigen-presenting cells (APCs) (4, 5). However, downregulation of these molecules is a common mechanism of immune evasion (6), albeit its correlation with tumour prognosis is not clear yet (7). For instance, NK cell infiltration has been associated with a lower risk of disease relapse in CRC (8). As a safeguard for T-cell function, NK cells control tumours that have reduced HLA-I expression and provide an activating balance of signals through surface receptors (9). Accordingly, NK cell-based immunotherapy is a promising alternative to conventional treatments and/or to the new immunotherapeutics based on T-cell activity.

Allogeneic adoptive NK cell transfer has been studied as a potential treatment for haematological malignancies and solid tumours over the last decades (10, 11). Although clinical trials have shown promising results against haematological malignancies (10, 12), its efficacy against solid tumours is controversial because of the need for migration and infiltration into a three-dimensional (3D) architecture (13, 14). Also, immunosuppressive mechanisms (i.e., HLA-I overexpression, TGF-β release, and the polarisation of tumour-associated macrophages) might negatively influence NK cell activity (15). As for the main immune checkpoints (ICs) involved in T-cell regulation such as CTLA-4, PD-1, LAG-3, or TIM-3, their role in regulating the anti-tumour ability of NK cells is not yet properly understood (16). Therefore, while the mechanism by which NK cells contribute to the efficacy of ICIs in cancer treatment is under evaluation (17–20), preliminary studies suggest that anti-tumoural activity of “healthy” NK cells might not be restricted by T cell-related ICs commonly expressed in the tumour microenvironment (TME). Thus, the role of these emergent ICs in the context of adoptive *in vitro* expanded NK cell transfer is still an important question to be addressed to optimise the anti-tumoural potential of NK cells to treat solid cancers like CRC.

CRC is a heterogeneous disease with challenges in therapy at both advanced and early stages. For the former, mutations in the KRAS/BRAF and PI3K/AKT pathways are commonly responsible for treatment failure and relapse after approved therapy treatment (21). For the latter, the use of adjuvant chemotherapy is controversial, as it has shown little benefit on overall survival. This is likely due to the use of traditional staging systems, which do not incorporate the effects of disease heterogeneity and the host immune response (22, 23). Further classification into different subtypes would allow us to better understand the mechanisms that determine clinical behaviour and to define personalised treatments (24). For instance, sporadic CRC can be classified into two types according to their genomic status and mutation abundance due to mismatch repair gene inactivation: microsatellite stable (MSS) and MSI tumours (25). In this line, a new classification of CRC tumours defined as consensus molecular subtype (CMS) was recently proposed (24). This classification considers the presence of specific molecular determinants in four main subtypes (CMS1-4). Each subtype differs in the immunological environment, survival prognosis, and response to therapy. While CMS2 and CMS3 tumours are poorly immunogenic, CMS1 and CMS4 differ in the type of immune infiltration. CMS1 tumours tend to accumulate a high number of mutations due to MSI status and attract immune effector cells, whereas CMS4 tumours exhibit an immunosuppressive TME with stromal infiltration (26). Despite that immunogenic tumours are susceptible to ICI therapies (3, 26), different immune evasion mechanisms such as HLA-I downmodulation, mutations in β2-microglobulin, loss-of-function mutations in JAK1/2, or WNT signalling activation can lead to acquired resistance to ICIs (27).

This landscape reveals new features of the CRC TME that influence disease progression and response to conventional treatments. This opens a niche for the implementation of novel therapeutic strategies where adoptive allogeneic NK cell therapy could provide positive results. Using transcriptomics and immunohistochemistry (IHC) data in a large cohort of CRC patients, we demonstrate a positive correlation between HLA-A downmodulation and survival in early-stage MSI tumours, which is associated with NK cell infiltration. Given this potentially protective role of NK cells, we have analysed the *in vitro* and *in vivo* efficacy of expanded allogeneic NK cells to eliminate CRC cells expressing mutations conferring chemoresistance and bad prognosis, as well as the effect of ICs on the anti-tumour ability of NK cells, as a basis for implementing allogenic adoptive NK cell transfer against difficult-to-treat CRC tumours.

## Material and Methods

### Ethical Statement

Human blood samples from healthy donors (HDs) were provided by the Blood and Tissue Bank of Aragón, integrated into the Spanish National Biobanks Network (PT20/00112) and processed following the Ethics and Scientific Committees procedures.

Animal experimental procedures were conducted according to the Federation of European Laboratory Animal Science Associations (FELASA). Protocols were approved by the University of Zaragoza’s Advisory Ethics Commission for Animal Research (P.I 47/18).

Colonomics series and CRC samples were used in IHC, written informed consent was obtained from all patients, and the Institution’s Ethics Committee authorised the protocol.

### Human Colorectal Cancer Samples

Our series, hereafter named Colonomics (CLX), includes gene expression data from 98 paired normal and stage II MSS tumour patients diagnosed at Bellvitge University Hospital (Colonomics project: www.colonomics.org; NCBI BioProject PRJNA188510). None of the patients received chemotherapy before the collection of the sample, and all of them have had a minimum follow-up of 3 years. In addition, public transcriptomic data from GSE39582 (n = 421), GSE13294 (n = 121), GSE14333 (n = 185), GSE17536 (n = 111), and The Cancer Genome Atlas (TCGA) (n = 143) series have been used. Their clinicopathologic features are described in **Supplementary Table 1**.

Also, for IHC staining, the “MSI series” comprising 36 patients with MSI colon cancer diagnosed at stage II at Bellvitge University Hospital was used. These samples were provided by the Biobank HUB-ICO-IDIBELL (PT17/0015/0024), integrated into the Spanish Biobank Network, and they were processed following standard operating procedures with the appropriate approval of the Ethics and Scientific Committees. Written informed consent was obtained from all patients, and the Institution’s Ethics Committee authorised the protocol.

### Consensus Molecular Subtype Classification and Microsatellite Instability Imputation

With the use of gene expression data, consensus gene expression signatures have been used to classify tumour samples into four different categories of CRC (CMS1, CMS2, CMS3 and CMS4) by applying the library CMS classifier in R. This method uses a Random Forest Approach to categorise a group of samples into different clusters of tumours.

Three out of the gene expression datasets had no information about the MSI/MSS status of the samples. Thus, missing MSI/MSS values were imputed using gene expression data. Datasets with MSI/MSS information were used to create a prediction model. The 50 most significant genes from each dataset were selected as predictors in the classification model. This list of genes was used in a classification model based on cross-validation (10-fold) based on K Nearest Neighbour (k = 5) to predict missing MSI/MSS values.

### HLA Immunohistochemistry Staining

Tissue samples of Colonomics series were cut with a microtome (4 µm) and deposited on adhesion slides. These sections were incubated for 15 min at 60°C to melt the paraffin, whereas the MSI series was incubated for 20 min because as they had been cut for a long time, the slides were immersed in paraffin for better preservation. After that, slides were deparaffinised. An indirect IHC technique was used. For antigen retrieval, the slides were boiled at 110°C in TRIS/EDTA buffer (Target Retrieval Solution, pH 9; Dako, Carpinteria, CA, USA) inside the Decloaking Chamber™ NxGen (BioCare Medical, Concord, CA, USA). Endogenous peroxidase was blocked for 5 min with 2% H_2_O_2_ followed by block buffer with 6% donkey serum. Thereafter, the primary antibody was incubated overnight at 4°C. The antibody used was rabbit anti-human HLA-A (1:500 dilutions; EP1395Y, GeneTex, Irvine, CA, USA). Excess of the primary antibody was washed with TBS 1× pH 7.4. For detection, slides were incubated with EnVision™+ Dual Link System-HRP (Dako) for 30 min at room temperature. The reaction was visualised using DAB (diluted in an imidazole-HCl buffer; DAB+ Kit, Dako) for 1 min 10 s. Finally, sections were counterstained with haematoxylin, dehydrated in increasing concentrations of ethanol, cleared with xylene, and mounted with Cytoseal™ 60 (Thermo Fisher Scientific, Waltham, MA, USA). Negative control was performed in the same conditions except for primary antibody absence. The stained slides were evaluated using a Leica DM600 microscope. Following the pathologist’s instructions (XS), a score of 1 to −2 was assigned for each sample, taking into account the intensity of HLA-A staining between tumour cells and stromal cells: i) 1 when the tumour cell was more intense than the stroma; ii) 0 when tumour cell and stroma had the same intensity; iii) −1 when tumour cell was less intense than the stroma; and iv) −2 when tumour cell was completely negative.

### Survival Analysis

HLA-A gene expression data were used to stratify patients between “High” and “Low” categories. The median value by the study was used as a cutoff. Disease-free survival (DFS) was plotted using a Kaplan–Meier curve and the Log-rank test was used to compare survival distributions between groups. Cox proportional hazards regression models were fitted to evaluate the prognostic value of HLA-A expression, adjusting by sex, age, and stromal infiltration and stratifying by study. Cox regression with Firth’s Personalized Likelihood was used in non-convergent models.

### Immune Cell Infiltration

To eliminate the batch effect, gene expression data from the 6 datasets were adjusted using the ComBat function from the R package SVA (28). The resulting expression matrix was used to infer the immune and stromal infiltrate. The R package microenvironment cell populations-counter (MCP-counter) (29) was used to quantify the relative fraction of a total of nine cell types, including seven immune and two stromal cell types. Moreover, a list of 18 gene markers from the R package Consensus TME (30) was used to perform enrichment analysis using the gene set variation analysis (GSVA) method (31). All comparisons between continuous variables were analysed using non-parametric tests (Wilcoxon test and Kruskal–Wallis test). For all tests applied, differences were considered statistically significant when the *p*-value <0.05. For the categorisation of HLA-A gene expression into “High” and “Low” groups, the median value was used as a cutoff.

### Cell Lines and Cultures

A panel of CRC cell lines, as well as LCL-EBV^+^ feeder cells, were cultured in basal medium (**Supplementary Table 2**) supplemented with 10% foetal bovine serum (FBS) (Sigma, St. Louis, MO, USA), 2 mM of ultraglutamine (Gibco, Grand Island, NY, USA), and 100 UI/ml of penicillin–0.1 mg/ml of streptomycin (Sigma-Aldrich). Cells were incubated at 37°C and 5% CO_2_.

Three-dimensional cultures of CRC cell lines were generated by the hanging drop method using methylcellulose, as described (32). Briefly, 25-µl droplets containing 1,000 cells/droplet were placed on the lid of a Petri dish filled with sterile water. The percentage of methylcellulose solution (Methocell) in the medium and the incubation time were adjusted for each cell line to favour the formation of spheroids (**Supplementary Table 2**).

Detachment of cells from plate surface and spheroid disaggregation was induced using Trypsin-EDTA (Sigma-Aldrich). Prior incubation of the spheroids in basal medium supplemented with 2 mM of EDTA promoted cell detachment and reduced the exposure time to Trypsin.

### NK Cell Activation

Peripheral blood mononuclear cells (PBMCs) from HDs were obtained by Ficoll gradient centrifugation (Sigma, Histopaque-1077). For NK cell *in vitro* activation, PBMCs were cultured in RPMI-1640 medium (Gibco) supplemented with 10% FBS (Sigma), 2 mM of ultraglutamine (Gibco), with the LCL-EBV+ R69 feeder cell line, at a 10:1 ratio (PBMCs:feeder cells) for 5 days, as described (33).

In order to establish a protocol to generate large numbers of NK cells, our previously developed activation protocol using LCL-R69^+^ as feeder cells was compared with the combination of these feeder cells and interleukins (100 UI/ml of IL-2, Miltenyi, Bergisch Gladbach, Germany; and 5 ng/ml of IL-15, Miltenyi). Cultures were maintained for 21 days, and stimuli and medium were renewed as explained: cell cultures were half diluted at day 7 of expansion, adding feeder cells and ILs according to PBMC number and final volume. From that time, and every 3 days, PBMC density was adjusted to 10^6^ cells/ml, and corresponding stimuli were incorporated. NK cell expansion rate was calculated by considering the number of PBMCs in culture and the percentage of NK cells at the beginning of the expansion and on days 7, 14, and 21 (analysed by flow cytometry).

To expand NK cells in the context of adoptive cell transfer in the mouse model, CD3^+^ cells were depleted from PBMCs before NK cell expansion. The combination of feeder cells and ILs was selected as the preferred stimuli to maintain the culture until the administration time. In all the experiments, NK cells were expanded for at least 14 days.

### Fluorescence-Activated Cell Sorting Characterisation

The phenotypic characterisation of NK cell receptors and their ligands in the CRC cell lines was analysed by flow cytometry (Beckman Gallios, Brea, CA, USA). Staining with labelled monoclonal antibodies was performed in phosphate-buffered saline (PBS) with 5% foetal calf serum (FCS) and 0.1% sodium azide, for 20 min at 4°C. Cells were then washed with PBS and fixed in 1% paraformaldehyde (PFA).

The following antibodies were used to label cell membrane receptors on the PBMC suspensions: CD3-FITC (clone BW264/56; Miltenyi), CD3-PerCP (clone BW264/56; Miltenyi), CD3-VioGreen (clone BW264/56; Miltenyi), CD56-PE (clone AF12-7H3; Miltenyi), CD56-APC (clone AF12-7H3; Miltenyi), CD56-PerCPVio700 (clone REA196; Miltenyi), CD16-FICT (clone REA423; Miltenyi), CD16-APC (clone VEP13; Miltenyi), NKG2A-PE (clone Z199; Beckman Coulter), NKp44-PE (clone 2.29; Miltenyi), CXCR3-PE (clone REA232; Miltenyi), PD-1-FITC (clone PD1.3.1.2; Miltenyi), TIM-3-APC (clone F38-2E2; Miltenyi), LAG-3-APC (REA351; Miltenyi), and CTLA-4-APC (clone BNI3; Miltenyi).

The expression of NK cell ligands on CRC cell lines was analysed in cell suspensions from both monolayer and spheroid cultures. The following antibodies were used for cell surface staining: ICAM-1-APC (clone HA58; BD, San Jose, CA, USA), HLA-ABC-FITC (clone W6/32; eBioscience, San Diego, CA, USA), HLA-E-PE (clone REA1031; Miltenyi), HLA-G-PerCPVio700 (clone 87G; Miltenyi), HLA-II-APCVio700 (REA332; Miltenyi), MICA/B-VioBright515 (clone REA1076; Miltenyi), PVR-PE (clone PV404.19; Miltenyi), Nectin-2-PE (clone R2.525; Miltenyi), ULBP-1-Alexa Fluor 405 (clone 170818; R&D, Minneapolis, MN, USA), ULBP-2,5,6-Alexa Fluor 405 (clone 165903; R&D), B7H6-Alexa Fluor 647 (clone 875002; R&D), PD-L1-APC (clone MIH1; eBioscience), PD-L2-PE (clone MIH18; Miltenyi), CEACAM-1-PeVio770 (clone TET2; Miltenyi), and Galectin-9-PE (clone REA435; Miltenyi). Galectin-9 was also analysed intracellularly, after fixation with 4% PFA for 15 min and subsequent permeabilisation with 1% saponin for 5 min.

Moreover, Annexin-V-FITC (Immunostep, Salamanca, Spain) was used to characterise phosphatidylserine (PS) basal level expression on the surface of CRC cells. In this case, Annexin Binding Buffer was used for cell staining.

### NK Cell Purification and Labelling

For *in vitro* experiments, activated NK cells were enriched from HD PBMCs by CD56-positive immunomagnetic separation (MACS, Miltenyi). NK cells were then fluorescently labelled with 3 µM of eFluor670 (eBioscience), following the manufacturer’s instructions.

For *in vivo* experiments, NK cells from freshly isolated PBMCs of HDs were enriched by CD3-negative immunomagnetic separation (MACS, Miltenyi) before NK cell expansion.

### Cytotoxicity Assays

Labelled NK cells were incubated with CRC cell lines at different effector:target (e:t) ratios and times at 37°C. For 2D assays, target cells were seeded in 96-multiwell flat-bottom plates 24 h prior to the 4 h of co-culture with NK cells. For 3D assays, 48–72-h-old formed spheroids were co-cultured with NK cells in 96-multiwell round-bottom plates for 48 h.

Subsequently, monocellular suspensions of cell cultures were obtained after Trypsin-EDTA disaggregation, and cell death was analysed by flow cytometry. Cell death markers, such as PS translocation and membrane permeabilisation, were monitored by Annexin-V and 7-Amino-Actinomycin (7-AAD) staining in the eFluor-negative target cell population.

### Checkpoint Blockade Experiments

Blocking monoclonal antibodies were used to analyse the impact of PD-1 and TIM-3 signalling on the anti-tumour response of activated NK cells. Considering the direct effect of ICIs on NK cell receptors, purified NK cells were incubated with 20 µg/ml of anti-PD1 (pembrolizumab) and/or 20 µg/ml of anti-TIM-3 (clone F382E2; BioLegend, San Diego, CA, USA) for 20 min at 4°C, before the co-culture with target cells. Human IgG1 isotype (Enzo, Farmingdale, NY, USA) measuring 5 µg/ml was used as control.

The efficacy of blockade was assessed by flow cytometry. Total PBMCs were incubated with the blocking antibodies before staining with the labelled flow cytometry antibody targeting PD-1 or TIM-3.

### NK Cell Transfer in a Xenograft Mouse Model

NK cells for adoptive cell transfer were obtained by initial depletion of CD3^+^ cells followed by an expansion protocol with the selected combination R69+IL-2+IL-15 for 14–21 days as described above. Enrichment of NK cells (CD56^+^CD3^−^) during the expansion was monitored by flow cytometry, with a final purity >85% in each cell culture. A pool of NK cells from 3 different donors was prepared before administration to avoid perturbations in results depending on HLA-I mismatch with the cell lines.

NOD-SCID IL2Rgamma^null^ (NSG) mice (female, 8 weeks old) were purchased from Charles River (Saint-Germain-Nuelles, France) and were housed in sterile facilities for immunosuppressed animals at the Centre for Biomedical Research of Aragon (CIBA).

The HCT-116 cell line was used to establish a xenograft CRC model. Thus, 10^6^ cells in 50 µl of Dulbecco’s modified Eagle medium (DMEM) were injected subcutaneously into the right flank, and mice were randomly divided into 3 groups: control, early treatment, and late treatment. Tumour development was analysed by measuring tumour volume (Volume = Width × Length × Height) every 2 days.

Early treatment started on day 5 after tumour induction when tumours reached a volume <50 mm^3^ but enough to be detected by palpation. Late treatment started on day 7 post tumour induction when tumours reached a volume of 50–100 mm^3^. Each mouse was administered up to three intraperitoneal (i.p.) injections of 10^7^ NK cells suspended in 100 µl of RPMI-1640 every 2 days. Treated and control groups received 10 µg/mouse of human recombinant IL-2.

Further experiments compared the efficacy of NK cell transfer alone or in combination with the anti-PD-1 monoclonal antibody pembrolizumab. This time, HCT-116 and DLD-1 cell lines were used to generate CRC xenograft models as described above. Mice were then randomly divided into 3 groups before receiving the therapy with the early treatment regimen: control, NK cells alone, or NK cells combined with pembrolizumab. Thus, treated mice received 10^7^ NK cells suspended in 100 µl of RPMI-1640 and 10 µg of IL-2 alone or in combination with 300 µg pembrolizumab/mouse. The control group receive only the RPMI-1640 vehicle with IL-2 and pembrolizumab.

Mice were monitored every 2 days and were sacrificed when they reached human endpoints as established by the Animal Ethics Committee (Volume larger than 1,500 mm^3^).

### Statistical Analysis of Target Cell Sensitivity to NK Cells

Correlation analyses were performed using R software. The correlation between NK cell ligand expression and the sensitivity of CRC cell lines to NK cells was analysed at low (1:1) and high (6:1) e:t ratios, in both 2D and 3D models. The non-parametric Spearman’s test was used due to the limited sample size and the inclusion of some variables that do not fit the normal distribution (Shapiro’s test). The correlation coefficient (r) and *p*-value were determined for each ligand and used to plot a correlation graph matrix. A simple regression model was then fitted with ligand expression level and percentage of cell death for each model and ratio. Ligands with a significant *p*-value were represented in a correlation plot. In addition, stepwise multivariate regression analysis tested the most significant ligands for univariate analysis.

GraphPad Prism (v5.0) software was used for further analysis. Depending on the characteristics of the experimental groups, Student’s t-test, one-way ANOVA, or two-way ANOVA was used as indicated. Survival curves were compared using the Log-rank test (Mantel–Cox). Statistical significance was always set at *p* < 0.05.

## Results

### Decreased HLA Expression Provides a Better Prognosis in Early Stages of Microsatellite Instability Colorectal Cancer and Correlates With NK Cell Infiltration

**Figure 1:**
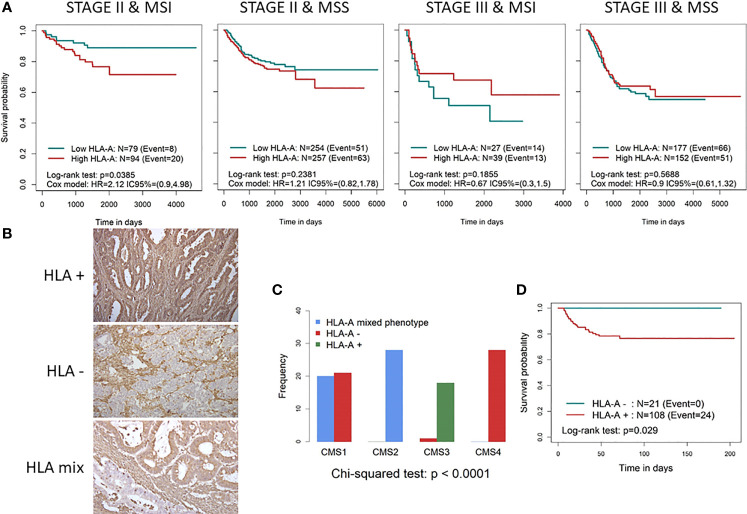
Characterisation of HLA-A expression as a prognostic biomarker in CRC patients. **(A)** Kaplan–Meier curves divided into high and low HLA-A categories in different CRC subtypes according to early/late stage and MSS/MSI status stratification. To define low (green line) and high (red line) HLA-A categories, the median gene expression was used as cutoff, in each dataset. p-Values were calculated using the binary Log-rank test and by fitting a Cox proportional hazards regression model stratifying by study and adjusted for age, sex, and stroma. **(B)** IHC staining of representative slides of tumours losing (total and partial) HLA expression (HLA^−^), tumours with HLA on surface (HLA^+^) and with a mixed phenotype (only part of the tumour with HLA lost). **(C)** Bar diagram showing the distribution of HLA^−^ (red), HLA mix (blue), and HLA^+^ (green) across the 4 CMS subtypes. **(D)** Kaplan–Meier curves divided into HLA^−^ (red) and HLA^+^ (blue) tumours in stage II MSI patients. CRC, colorectal cancer; MSS, microsatellite stable; MSI, microsatellite instability; IHC, immunohistochemistry; CMS, consensus molecular subtype.

Immune-related markers were analysed in samples from CRC patients. We found a better prognosis for patients with MSI stage II CRC, but not MSS, who presented HLA-A mRNA downregulation (**Figure 1A**). This effect was lost at stage III (late stage; **Figure 1A**), which could be explained by tumour immunosuppressive mechanisms responsible for cancer progression and immune failure. Indeed, as indicated below, MSI tumours presented increased NK cell infiltration that correlated with HLA expression in low-stage tumours, suggesting that immunological status as well as advanced stages restrict NK cell infiltration and, thus, their protective role in CRC development. Therefore, we stratified CRC samples by CMS and found that HLA-A mRNA downregulation is a good prognostic biomarker only in stage II CMS1 samples (**Supplementary Figure 1**). Again, this effect did not correlate with a better prognosis in any of the CMS subgroups at stage III.

Similar results were obtained for the other MHC class I genes HLA-B and HLA-C (**Supplementary Figure 2**), suggesting that the immune system plays a role in controlling hypermutated MSI/CMS1 tumours in the early stages of the disease.

Since the analysis of HLA mRNA expression does not always predict the level of protein expression, we decided to confirm our results by analysing the expression of HLA-I at the protein level by IHC in order to validate the association between HLA downmodulation and better survival in stage II MSI patients. To this end, 134 samples from stage II CRC patients were analysed by IHC. All nucleated cells showed positive HLA-A staining (**Figure 1B**), which indicated the reliability of the results. Two of the samples were excluded for further analysis since they were found to be negative for the presence of tumour cells. Total loss of HLA-A was observed in 14 CRC samples (10.6%), partial loss was observed in 7 samples (5.3%), and a mixed positive and negative expression of HLA-A was identified in 3 samples (**Figures 1B, C**).

As expected, due to HLA-A loss and MSI status association, significant differences were observed across the CMS subtypes (*p*-value <0.001, **Figure 1C**). Only CMS1 tumours as well as one CMS3 tumour lost HLA-A expression. Of note, the mixed HLA-A score was only represented by CMS1 and CMS3 subtypes. As shown by the Kaplan–Meier curves (**Figure 1D**), a clear association between HLA-A loss and survival was observed. None of the 21 patients in the HLA-A-negative group (20 MSI patients) experienced disease relapse (*p*-value = 0.026). In this case, the mixed phenotype was excluded from the analysis due to the small number of samples.

**Figure 2:**
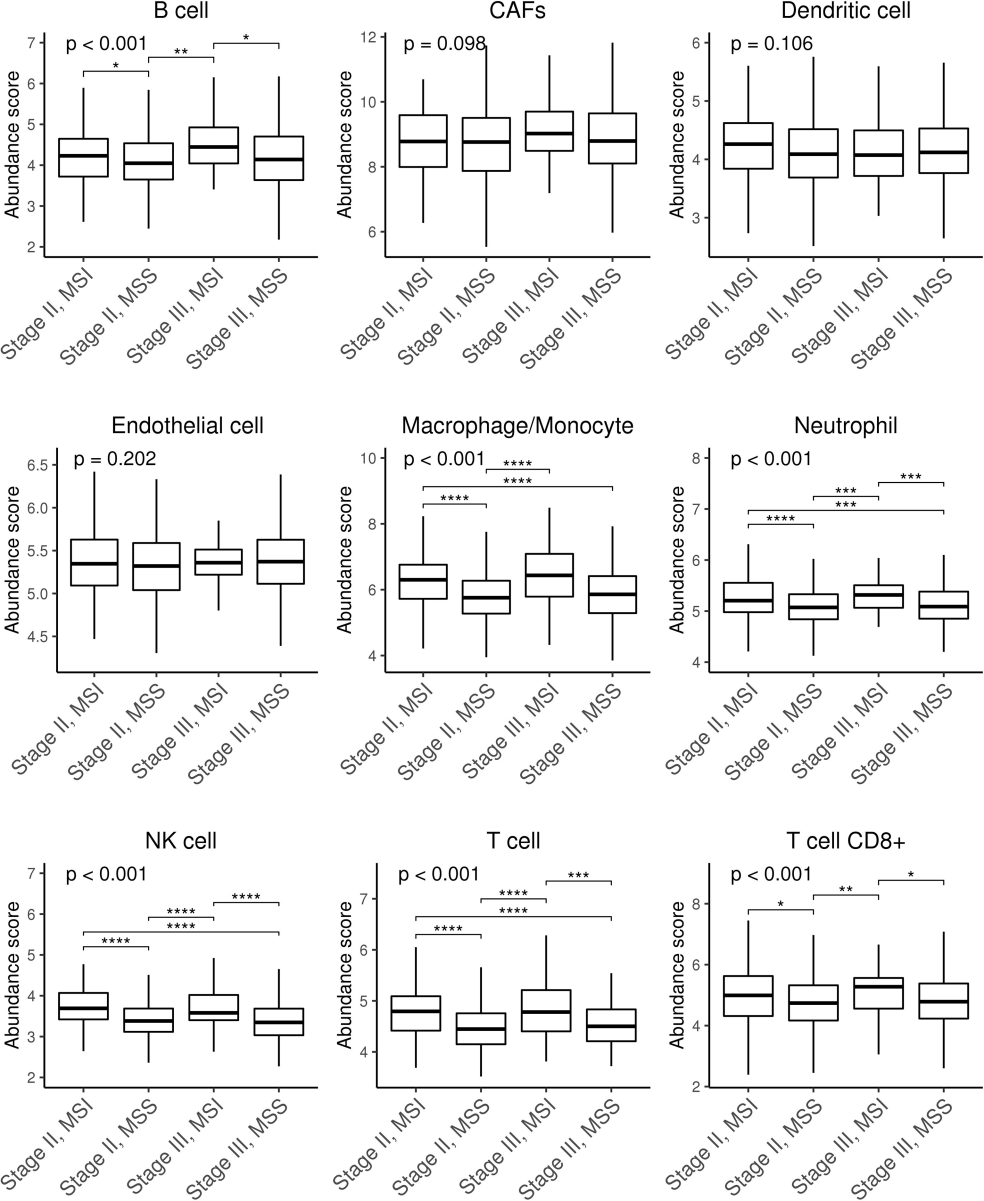
Box plots showing stromal and immune infiltration across CRC patients stratified by stage and MSS/MSI status. Stromal (CAFs and endothelial) and immune (B cells, dendritic cells, macrophages/monocytes, neutrophils, NK cells, T cells, and CD8^+^ T cells) infiltration scores were calculated using microenvironment cell populations-counter (MCP-counter) tool. Differences were assessed using non-parametric Kruskal–Wallis and Tukey’s tests (*p ≤ 0.05; **p ≤ 0.01; ***p ≤ 0.001; ****p ≤ 0. 0001). CAFs, cancer-associated fibroblasts; NK, natural killer; CRC, colorectal cancer; MSS, microsatellite stable; MSI, microsatellite instability.

We next wondered whether differences in immune cell infiltration could explain this phenotype. **Figure 2** shows differences in macrophages, neutrophils, NK cells, and T cells in MSI *vs*. MSS tumours and between stages II and III, and also in B cells between stage II and stage III. As expected, MSI tumours were more infiltrated than MSS. Similar results were obtained when a different tool was used (**Supplementary Figure 3**). Therefore, we confirmed that CMS1 and CMS4 subtypes had higher levels of immune and stromal infiltration than CMS2-3 subtypes in all our series. Consistent with all datasets, CMS4 samples had more fibroblasts and endothelial cells (**Supplementary Figure 3**).

**Figure 3:**
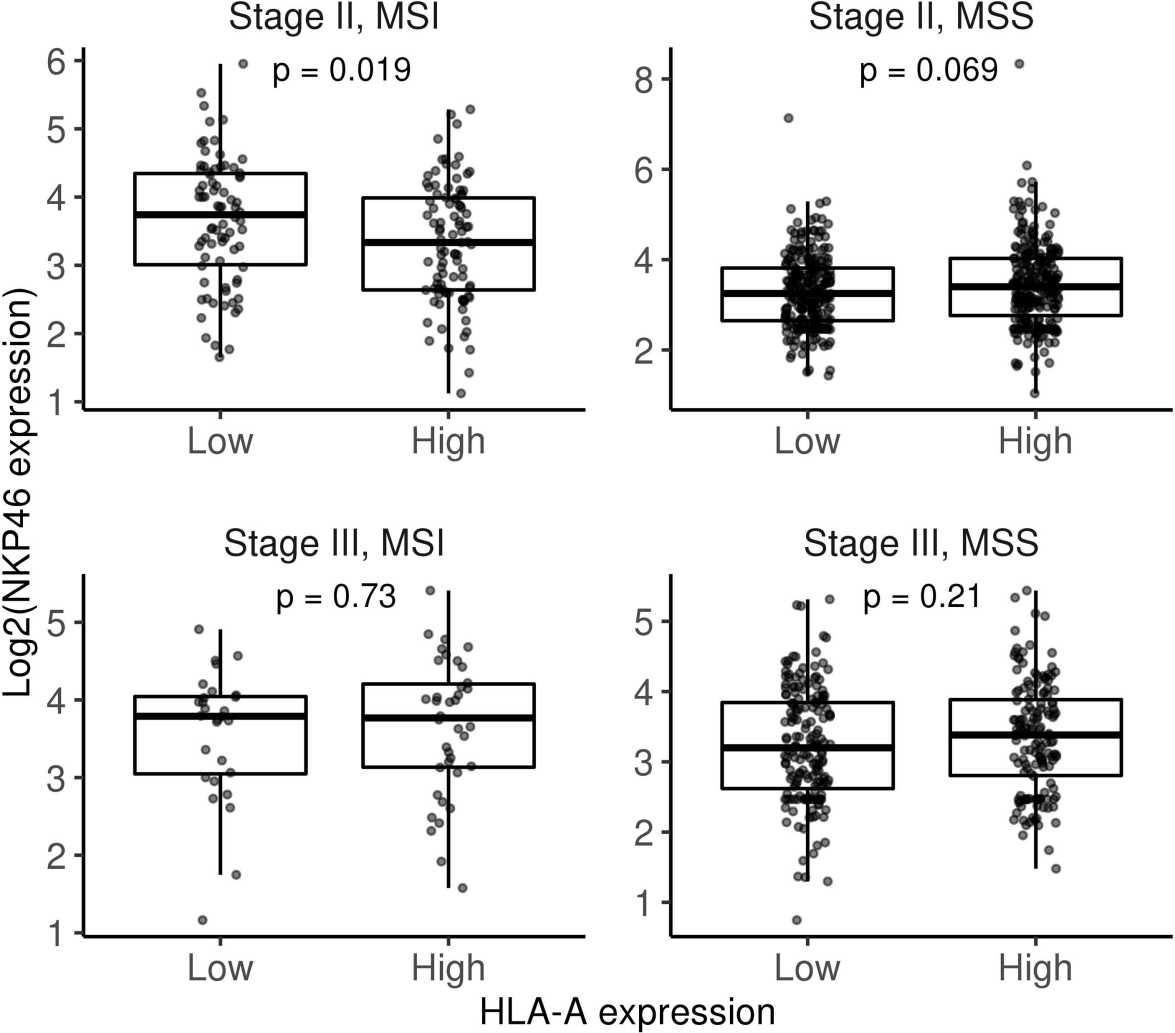
Box plots comparing NKp46 gene expression between low and high HLA-A categories (gene expression median was used as a cutoff) stratifying by stage and MSI/MSS status. Differences were assessed using non-parametric Wilcoxon test. MSI, microsatellite instability; MSS, microsatellite stable.

We hypothesise that tumours lacking HLA, and therefore exhibiting a self-missing phenotype, could be recognised by NK cells. We found no differences between stage II and stage III MSI tumours in terms of NK infiltration based on a gene signature score (**Figure 2**). However, as shown in **Figure 3**, detection of the NK cell marker, NKp46/NCR1, which to date is the most reliable marker for NK cell detection, revealed higher NK cell levels in stage II MSI tumours without HLA expression. Meanwhile, no differences in NKp46 expression were found between low or high HLA expression in MSI stage III tumours or MSS tumours at any stage. These results suggest that NK cells play a role in controlling immune-infiltrated HLA-deficient tumours before they progress and that they might be influenced by different immunosuppressive mechanisms. Thus, adoptive NK cell transfer could be considered a potential treatment for those HLA-I-negative tumours that are refractory to ICI-based immunotherapy and might play a role in eliminating less immunogenic tumours.

### Activated NK Cells Are Able to Kill Colorectal Cancer Cell Lines That Correlate With HLA-I Expression

**Figure 4:**
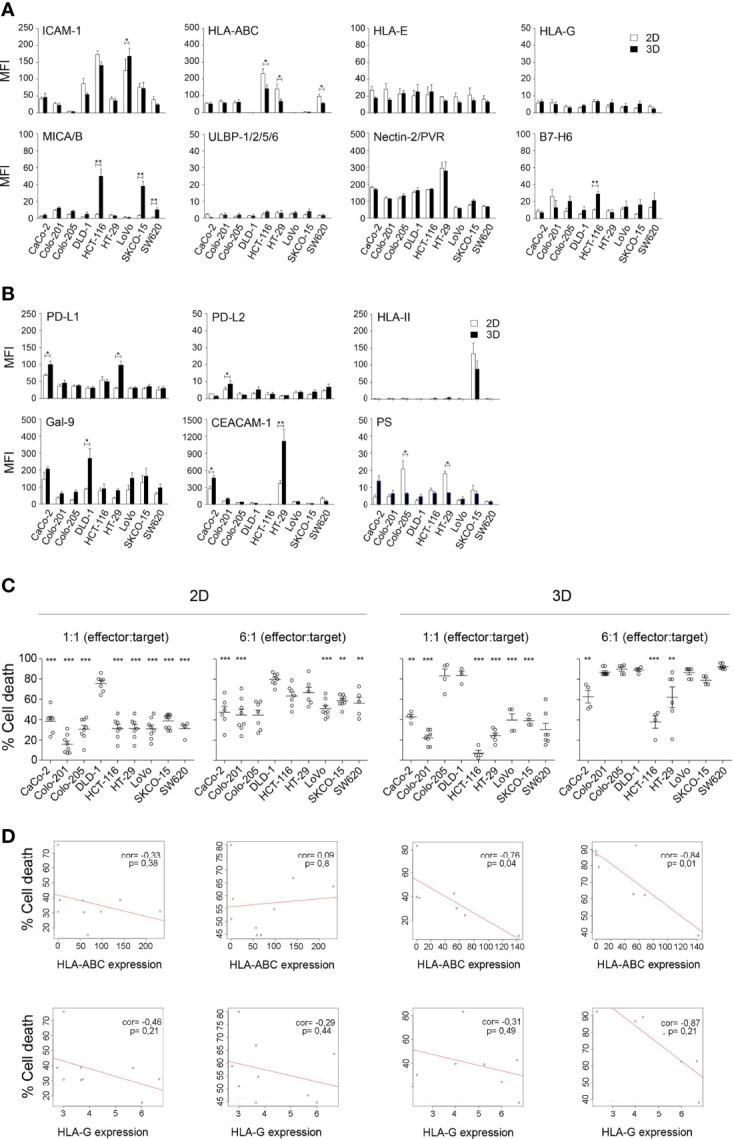
Phenotypic characterisation of CRC cell lines and their correlation with sensitivity to activated NK cells. **(A)** Expression of ligands for conventional NK cell checkpoints in CRC cell lines cultured in 2D and 3D conditions. Data are presented as mean ± SEM of at least 4 experiments. Statistical analyses were performed by t-test. ^∗^ *p* < 0.05; ^∗∗^ *p* < 0.01. **(B)** Expression of ligands for emerging NK cell checkpoints in CRC cell lines cultured in 2D and 3D conditions. Data are presented as mean ± SEM of at least 4 experiments. Statistical analyses were performed by t-test. ^∗^ *p* < 0.05; ^∗∗^ *p* < 0.01. **(C)** Cytotoxicity of activated NK cells against a panel of CRC cell lines in both 2D and 3D conditions. Cell death of CRC cells was analysed after 4 h co-culture (2D) or 48 h co-culture (3D) with activated NK cells, at low (3:1) and high (6:1) e:t ratios, by flow cytometry using Annexin-V/PS staining in the e-Fluor670-negative population. Data are presented as mean ± SEM from at least 4 donors after subtraction of respective controls without effector NK cells. Cell viability without NK cells was always >85%. Statistical analyses were performed by one-way ANOVA test with Dunnett’s multiple-comparison post-test. ^∗∗^ *p* < 0.01; ^∗∗∗^ *p* < 0.001. **(D)** Correlation between HLA-ABC or HLA-G expression and cell death induced by activated NK cells in CRC cell lines. A simple regression model was then fitted with ligand expression level and percentage of cell death for both low and high e:t ratios and 2D and 3D cultures. Plots represent ligands with a significant *p*-value. ^∗^ *p* < 0.05. CRC, colorectal cancer.

To study the ability of allogeneic activated NK cells to eliminate different types of CRC, we established a CRC cell line collection with different mutational statuses on the EGFR pathway (Ras/Raf; PI3K/AKT) and p53 (**Supplementary Table 3**). Our aim was to have a representative model of the molecular heterogeneity of CRC and to analyse the suitability of 3D spheroid models to mimic the *in vivo* CRC architecture (**Figure 4**, **Supplementary Figure 4**). We analysed ligands for both conventional NK cell checkpoints (**Figure 4A**), which have been shown to affect NK cell activity, and emerging checkpoints (**Figure 4B**), which are known to modulate T-cell activity but whose effects on NK cells have not been well established yet (16). Remarkably, the Colo201 and Colo205 cell lines expressed low levels of ICAM-1, an essential molecule for immunological synapse formation. In addition, DLD-1, LoVo, and SKCO-15 cell lines lack HLA-ABC, the major inhibitory molecule for NK cells. Other ligands showed a variable expression pattern in the different cell lines. However, notably, 3D conformation was associated with changes in the expression of these molecules, which could affect the cytotoxicity of NK cells. Although the fluctuation pattern of the ligands depended on the cell line, ICAM-1, HLA-ABC, and PS were among the ligands with a decreased expression in 3D conditions, whereas MICA/B, PDL-1, and CEACAM-1 were increased in the 3D model (**Figures 4A, B**).

To determine whether these changes had an impact on the sensitivity of the CRC cell lines to activated NK cells, we performed cytotoxicity assays at low (1:1) and high (6:1) (e:t) ratios under both 2D and 3D conditions. We used NK cells activated following a protocol previously established in our group combining the LCL-EBV^+^ R69 feeder cell line (33) with cytokines (IL-2 and IL-15) that promoted NK cell expansion (**Supplementary Figure 5**). The population of activated NK (eNK) cells obtained with this protocol is characterised by a high expression of CD56 and CD16 and a high cytotoxic activity that did not change from day 5 to day 21 of expansion (**Supplementary Figure 5**). Regarding the expression of other receptors involved in NK cell activity, activated NK upregulated NKp44, NKG2A, and CXCR3 receptors, confirming the activated phenotype. Concerning IC expression, activated NK cells expressed high levels of TIM3; meanwhile, the level of PD1 and CTLA4 remained low, and LAG3 expression was induced during the expansion of a population of NK cells (**Supplementary Figure 6**).

As shown in **Figure 4C**, the DLD-1 cell line was the most sensitive to eNK cells in both 2D and 3D cultures, followed by HCT-116 and HT-29 at a high e:t ratio under 2D conditions. Remarkably, the results were different in 3D cell cultures, and both cell lines became resistant when a 3D structure was formed, with HCT-116 being the most resistant cell line. These results could be related not only to the expression level of the different NK cell ligands but also to the spheroid compaction. Indeed, the Colo205 cell line was unable to form a compact spheroid and proved to be one of the most sensitive cell lines in 3D conditions. Consequently, we performed a multivariate regression analysis to analyse the correlation between the ligand expression levels and the sensitivity of the CRC cell lines to NK cells. A negative correlation was found between sensitivity to NK cells and the levels of the inhibitory ligands HLA-ABC and HLA-G (**Figure 4D**, and **Supplementary Figures 4** and **7**). Moreover, this correlation became significant when the CRC cell lines were cultured in the 3D model, for both HLA-ABC and HLA-G, demonstrating the greater robustness of the 3D model to mimic physiological conditions.

These results indicate that the efficacy of adoptive NK cell therapy is influenced by HLA-I expression *in vitro*. Nevertheless, activated NK cells still showed cytotoxic activity against tumour cells with high HLA-I expression regardless of the expression of ligands for ICs or the presence of mutations in various signalling pathways. This effect is likely due to the use of allogeneic NK cells, as has been shown previously in haematological tumours (34), although HLA mismatch was not determined.

### The Combination of Allogenic eNK Cells With IL-2 Delay Colorectal Cancer Xenograft Development *In Vivo*, Which Is Not Improved by Including the PD-1 Blocking Antibody Pembrolizumab

**Figure 5:**
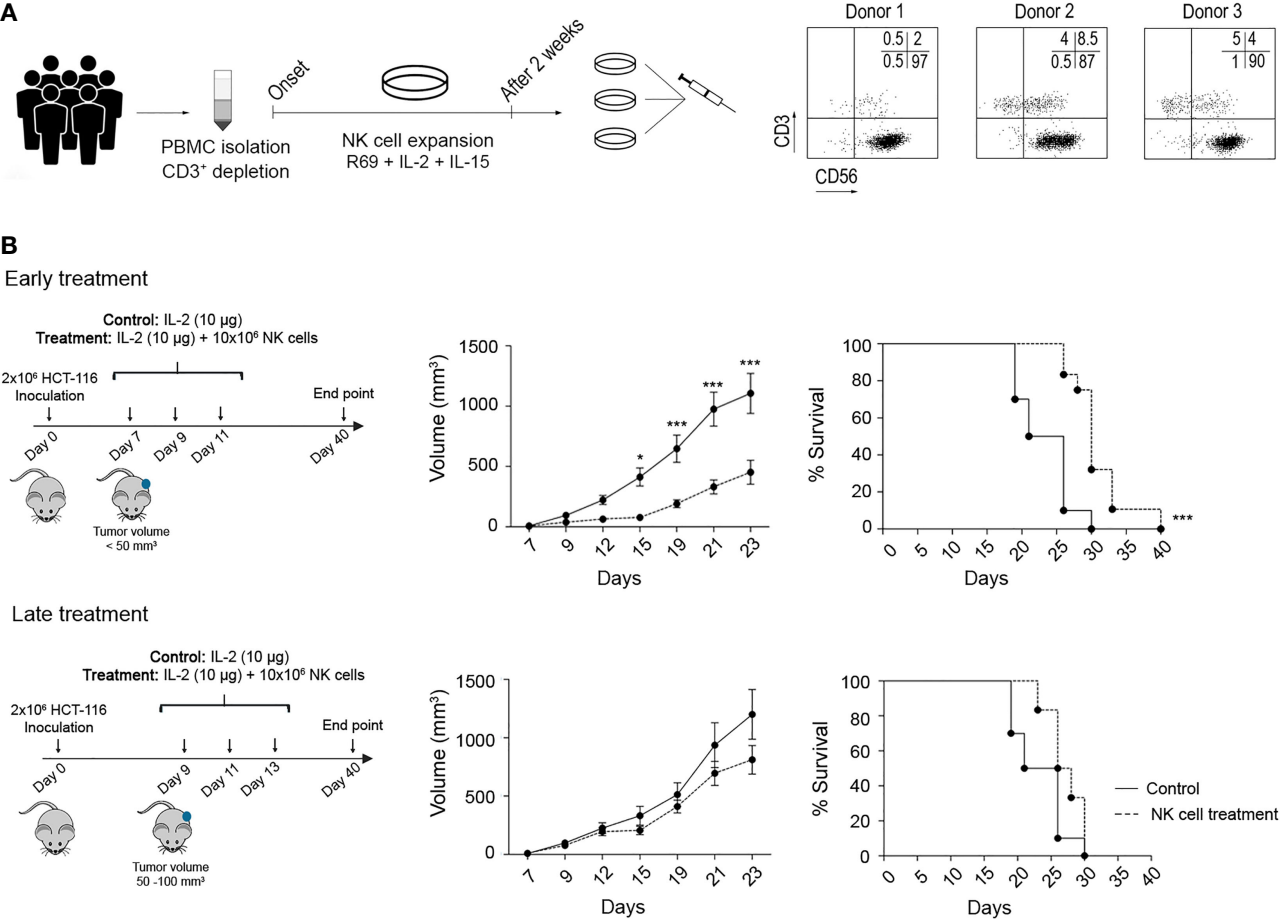
Efficacy of adoptive NK cell transfer against CRC tumours in a xenograft model. **(A)** NK cell expansion protocol for adoptive transfer. PBMCs derived from six HDs were depleted from the CD3^+^ population and expanded for 14 days with LCL-EVB+R69 feeder cells (1:10 feeder:NK cells), IL-2 (100 UI/ml), and IL-15 (5 ng/ml). The NK cell purity for each donor was analysed by flow cytometry before preparing a pool of NK cells combining 3 different cell cultures. **(B)** Efficacy of activated NK cells against HCT-116 tumours after an early or a late treatment. On day 0, NSG mice were inoculated with 2 × 10^6^ HCT-116 cells. After 5 (early treatment) or 7 (late treatment) days, mice were i.p. inoculated with vehicle (RPMI and 10 µg/mouse IL-2) or treatment (10^7^ NK cells and 10 µg/mouse IL-2). Tumour development was monitored over 40 days as described in *Material and Methods*. Data are presented as mean ± SEM of 10 mice in 2 independent experiments for control and early treatment groups, and 6 mice in 1 experiment for late treatment group. Statistical analyses to compare tumour volume curves were performed by two-way ANOVA test with Bonferroni’s post-test. Survival curves were analysed with Log-rank (Mantel–Cox) test. ^∗^ *p* < 0.05; ^∗∗∗^ *p* < 0.001. CRC, colorectal cancer; PBMCs, peripheral blood mononuclear cells; HDs, healthy donors; RPMI, Roswell Park Memorial Institute.

Finally, we analysed the efficacy of adoptive NK cell transfer *in vivo*. HCT-116 and DLD-1 cell lines were inoculated to generate a xenograft model. As described above, they respectively correspond with the most resistant and sensitive cell lines to NK cell cytotoxicity in 3D cultures. For each experiment, NK cells from different donors were simultaneously expanded for 2 weeks after CD3^+^ T-cell depletion (see *Material and Methods*, **Figure 5A**). Then, a pool of NK cells was prepared to reduce the effect of donor allogenicity. The purity of NK cells was analysed by flow cytometry before the transfer, with acceptance values above 85% (**Figure 5A**).

NSG mice were inoculated with 2 × 10^6^ HCT-116 cells and distributed into 3 different groups: control, early treatment (5 days post-inoculation and tumour volume <50 mm^3^), or late treatment (7 days post-inoculation and tumour volume 50–100 mm^3^). According to the treatment schedule, mice were i.p. inoculated with vehicle (media and IL-2) or 10^6^ NK cells and IL-2 (**Figure 5B**). As shown, a significant delay in tumour growth was observed in the early treatment group, while the late treatment could not restrain tumour progression. This was translated into a significantly prolonged survival for the early treatment group. These results prove that eNK cells can control tumour development at an early stage. At late stages, both the tumour volume and tumour-induced immunosuppressive mechanisms can dampen NK cell function, in agreement with our observations in CRC patient samples.

**Figure 6:**
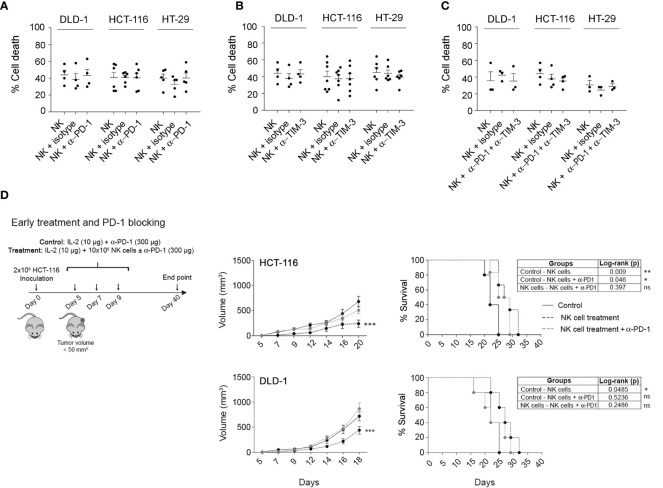
Combination of activated NK cells and PD-1 and TIM-3 checkpoint blockers against CRC cell lines. **(A–C)** *In vitro* cytotoxicity of activated NK cells in the presence of anti-PD-1 **(A)**, anti-TIM-3 **(B)**, or both antibodies **(C)**. Cell death of CRC cells was analysed after 4 h co-culture (2D) with activated NK cells alone, with 20 µg/ml of pembrolizumab and/or anti-TIM-3, or 5 µg/ml of IgG isotype, at 0.5:1 (e:t) for DLD-1 or 1:1 (e:t) for HCT-116 or HT-29, by flow cytometry using Annexin-V/PS staining in the e-Fluor670-negative population. Data are presented as mean ± SEM from at least 3 donors after subtraction of respective controls without effector NK cells. Cell viability without NK cells was always >85%. Statistical analyses were performed by one-way ANOVA test with Bonferroni’s post-test. **(D)** Efficacy of eNK cells, alone or in combination with pembrolizumab, against HCT-116 or DLD-1 xenograft tumours after an early treatment. On day 0, NSG mice were inoculated with 2 × 10^6^ HCT-116 or DLD-1 cells. After 5 (early treatment), mice were i.p. inoculated with vehicle (RPMI and 10 µg/mouse IL-2), NK cells alone (10^7^ NK cells and 10 µg/mouse IL-2), or NK cells with 300 µg/mouse pembrolizumab. Data are presented as mean ± SEM of 6 mice in 1 experiment. Statistical analyses to compare tumour volume curves were performed by two-way ANOVA test with Bonferroni’s post-test. Survival curves were analysed with Log-rank (Mantel-Cox) test. ^∗^ *p* < 0.05; ^∗∗^ *p* < 0.01; ^∗∗∗^ *p* < 0.001. NS, Not Statistically Significant. CRC, colorectal cancer; RPMI, Roswell Park Memorial Institute.

One study has previously reported in cancer mouse models that the efficacy of PD-1/PDL-1 inhibitors partly depends on the presence of NK cells (17). Although NK cells activated with our protocol showed low PD-1 expression, it might be possible that PD-1 expression is regulated by other factors derived from tumour cells and/or from soluble or cellular components of the TME. Thus, we analysed the impact of PD-1 blockade on the efficacy of allogeneic adoptive NK cell transfer *in vitro* and *in vivo.* First, as shown in **Supplementary Figure 8A**, we confirmed that pembrolizumab binds to PD-1 as it completely prevented the staining with another fluorescently labelled anti-PD-1 antibody. Next, the cytotoxicity of activated NK cells in combination with pembrolizumab was analysed *in vitro* against HCT-116, HT-29, and DLD-1 CRC cell lines (**Figure 6A**), which showed differences in PD-L1 expression. In any case, the addition of pembrolizumab or IgG isotype resulted in an increase in NK cell cytotoxicity. As NK cells expressed high levels of TIM-3, we also tested a TIM-3 blocking antibody alone or in combination with the anti-PD-1 antibody. As shown in **Supplementary Figure 8B**, we were equally able to confirm that anti-TIM-3 blocking antibodies bound to target cells. However, TIM-3 blockade alone or in combination with pembrolizumab did not enhance NK cell cytotoxicity (**Figures 6B, C**).

Finally, we analysed the effect of the combination of allogeneic adoptive eNK cells, IL-2, and pembrolizumab in the control of tumour growth in HCT-116 and DLD-1 cells. In contrast to the *in vitro* models, eNK cells in combination with IL-2 significantly and similarly delayed tumour growth in both cell lines. Furthermore, we did not find better outcomes when pembrolizumab was administered together with the eNK cells (**Figure 6D**). Strikingly, pembrolizumab reduced the efficacy of adoptive eNK cells, which suggests that pembrolizumab dampens NK cell activity or promotes tumour cell growth.

## Discussion

Reactivating patient immunity with antibodies targeting T cell-related ICs is a revolutionary approach that has changed the treatment paradigm and survival, reaching durable responses (1). However, only a small proportion of tumours are sensitive to ICIs, and other alternatives need to be considered. Here we have analysed adoptive NK cell transfer as an alternative for CRC tumours harbouring mutations that confer a bad prognosis. As a basis, our study suggests that NK cells control CRC development as HLA-I downregulation correlates with higher NK cell infiltration and better prognosis in stage II MSI CRC patients, including reduced recurrence and metastasis. In contrast, in advanced stage III CRC patients as well as in stage II MSS patients, this correlation is most likely due to the reduced NK cell infiltration found in these tumours. Our *in vitro* and *in vivo* results show that large-scale allogeneic eNK cells eliminate MSI and MSS CRC tumours as well as bad prognosis CRC cells that do not respond to chemotherapy or antibody-mediated immunotherapy treatments, irrespective of the presence of some T cell-related ICIs. Thus, altogether, these findings strongly support the use of allogeneic adoptive NK cell transfer to treat CRC tumours, especially those tumours in which tumour cells might have immune-escaped from host NK cells. In addition, allogeneic NK cells might be a good option as adjuvant therapy to prevent recurrence and metastasis in stage II CRC patients after surgery.

CRC is a solid tumour characterised by a high degree of heterogeneity associated with the accumulation of a variety of mutations that determine response to treatments and survival (24). This heterogeneity also affects the TME and the *Immunoscore* used to classify CRC tumours (35). Even if HLA molecules are key components in triggering an anti-tumour T-cell response, the prognostic value of HLA is unclear (7). HLA-I downmodulation has been described as a common immune evasion mechanism, especially relevant for resistance to conventional T cell-related ICIs resistance, in MSI but not MSS tumours (36). In contrast, there is evidence that some patients with HLA-I downmodulation have a better prognosis (7, 37). This discrepancy could be explained by the lack of data on CMS or MSI/MSS classification. Here we demonstrate that HLA-A downmodulation correlates a with better prognosis in MSI stage II tumours, but this effect is lost in MSS tumours. Furthermore, this phenotype was exclusive to CMS1 tumours and was associated with non-metastatic disease. This finding is supported by previous results in mouse models indicating that NK cells are involved in the control of tumour metastasis (38). Indeed, our results show that no tumour that lost HLA-A in its membrane experienced a relapse. Menon et al. also described that HLA-A but not HLA-B/C downmodulation correlates with longer DFS in patients with low-stage CRC, suggesting an anti-tumour role of NK cells or attenuated aggressiveness of MSI tumours (37). We found that HLA-A downmodulation in stage III MSI tumours does not correlate with better prognosis, suggesting the development of immune escape mechanisms. Indeed, stage III MSI CRC exhibited lower NK cell infiltration, which could explain the lack of correlation between HLA-A and prognosis.

In this context, our analysis of immune infiltration in CRC showed a higher increase in the NK activation marker *NKP46*, the most specific marker to detect NK cells, in MSI stage II tumours with reduced HLA-A. Of note, the use of IHC in the study to assess the expression of membrane HLA-A provided more reliable results than those obtained by mRNA techniques, as RNA extraction can also capture the molecules derived from the stroma and infiltrated immune cells (39). Albeit more MSI/CMS1 patients who relapse are needed to obtain a more significant prognostic value of HLA-A, it could be concluded that the lack of expression of membrane-associated HLA-A on the tumour is a good prognostic factor in CMS1 stage II CRC patients. Together with previous observations showing a correlation between NK cell infiltration and CRC prognosis (40, 41), these results support the implication of NK cells in controlling tumour development and the use of adoptive NK cell transfer in those cases where tumour cells have avoided the host NK cell response.

Thus, we developed an NK cell-based therapy that could overcome the mechanisms of tumour immune escape and poor prognosis based on a protocol previously established in our lab employing the LCL-EBV^+^ R69 feeder cell line (33), which generates activated NK cells able to efficiently eliminate drug-resistant haematological cancer cells (34) as well as to migrate and destroy conventional and 3D CRC cell cultures (14, 32). Remarkably, PD1 was almost absent from these activated NK cells, which supports our findings showing that anti-PD-1 antibodies do not improve the response of adoptive NK cell transfer *in vitro* or *in vivo* using eNK cells from HDs, suggesting that eNK cells might overcome the expression of PD-1 ligands in the TME. This result contrasts with the high levels of PD-1 observed in NK cells from cancer patients, which may explain the contribution of PD1 to the regulation of the anti-tumoural NK cell activity in cancer patients (42, 43).

Remarkably we have demonstrated that the acquisition of a 3D conformation can alter the sensitivity of cell lines to NK cells, because of both phenotypic alterations, as observed for some NK cell ligands, and the degree of compaction as described elsewhere (44). In addition, the finding that the anti-tumour effect of activated NK cells is restricted by HLA-ABC and HLA-G only in the 3D model proves its biological relevance. This result agrees with the observation that NK cell infiltration correlates with a good prognosis only in patients with low expression of HLA-A. Supporting our results, some studies have found HLA-G overexpression in CRC patients with poor prognosis (45). Nevertheless, irrespective of HLA-I expression, activated NK cells could still eliminate CRC spheroids at a high e:t ratio, opening the chance to implement NK cell adoptive therapy against CRC. Whether this effect is due to HLA-KIR mismatch as seen in haematological malignancies will require further experimental work.

The relevance of the results in the *in vitro* model was evaluated in a xenograft model using NSG mice. Although the use of this model limits the study of the TME, it still allowed us to analyse NK cell migration, the infiltration into the extracellular matrix, and the interaction of the NK cells with the tumour and some populations of the TME, such as fibroblasts and endothelial cells, including potential immunosuppressive factors. Adoptive NK cell transfer retarded tumour growth and improved survival when administered early after tumour development. However, late treatment failed to control tumour growth, probably due to a direct impact of the tumour size or alterations in TME during tumour development. In contrast to the *in vitro* models, eNK cells similarly delayed tumour growth HLA-I positive (HCT-116) and negative (DLD-1) cell lines *in vivo*. This could be explained by a higher growth rate of DLD-1 cells *in vivo* as previously described (46). As indicated above, the *in vivo* efficacy of early administration of eNK cells was not improved by the anti-PD-1 mAb pembrolizumab irrespective of HLA-I expression. Strikingly, pembrolizumab reduced the efficacy of eNK cells, which somehow indicates that pembrolizumab either reduces NK cell activity or promotes tumour cell growth. Lenaro et al. found that the engagement of the anti-PD-1 antibody nivolumab to PD-1 protected CRC cells from chemotherapy-induced cell death. However, as we did not find an accelerated development of control HCT-116 tumours in the presence (**Figure 6D**) or absence (**Figure 5B**) of pembrolizumab, our results suggest that pembrolizumab somehow affects NK cell activity *in vivo*. Further studies will be required to confirm if PD-1 blocking has any potential negative effect on adoptive NK cell therapy success and the potential mechanism(s) involved.

Our findings indicate that activated NK cells eliminate CRC cells *in vitro* and *in vivo* irrespectively of the mutational status of pathways involved in treatment resistance, and the results in human samples suggest that NK cells play a key role in the control of early-stage MSI CRC tumours, leading to fewer metastases and better prognosis; the latter further supported by the finding that loss of HLA is a good prognosis biomarker in stage II MSI tumours. Thus, our results present potential implications for both patient stratification and therapy. On the one hand, we conclude that tumours in the early stages of the disease losing HLA as a mechanism of immune escape are likely more prone to NK cell attack and subsequently exhibit a better prognosis. On the other hand, tumours that have managed to avoid NK cell-mediated immunosurveillance presenting low NK cell infiltration are candidates for NK cell adoptive therapy, as activated allogeneic NK cells, albeit restricted by HLA-I expression, are still able to eliminate HLA-I-positive tumours including those presenting bad prognosis status and independently of the presence of T cell-related inhibitory IC ligands. Thus, this study, combining results from CRC patients with functional *in vitro* and *in vivo* studies with eNK cells, provides the molecular basis to support the development of clinical trials to treat CRC using expanded allogeneic NK cells.

## Data Availability Statement

The datasets presented in this study can be found in online repositories. The names of the repository/repositories and accession number(s) can be found below: https://www.ncbi.nlm.nih.gov/, BioProject PRJNA188510.

## Ethics Statement

The studies involving human participants were reviewed and approved by the Blood and Tissue Bank of Aragón (PT20/00112) and Biobank HUB-ICO-IDIBELL (PT17/0015/0024) integrated into the Spanish National Biobanks Network. The patients/participants provided their written informed consent to participate in this study. The animal study was reviewed and approved by the University of Zaragoza’s Advisory Ethics Commission for Animal Research (P.I 47/18).

## Author Contributions

PM, MA, RS-P, and JP were involved in the conception, design, and preparation of the manuscript. PM and MA were equally involved in the research, analysis, and interpretation of the results. MA, SG-M, and RS-P performed the computer data analysis. CS, XS, RP-C, and MA-F supervised the patients, biopsied tissues, and pharmacological treatments. SH, SG-M, RA, LS, LC, PJ-S, CP, and AR-L participated in the *in vitro* experiments and contributed to the interpretation of the results. SR participated in *in vivo* experiments. SG-M, IU-M, and EG contributed to the interpretation of the results and revised the manuscript. MA, RS-P, and JP contributed equally to the development of the theoretical framework and project management and supervision. All authors read and approved the final manuscript.

## Funding

Work in the JP laboratory is funded by ASPANOA, CIBER (CB 2021; Instituto de Salud Carlos III, Ministerio de Ciencia, Innovación and Union Europea.NextGenerationEU), Fundacion Inocente, Carrera de la Mujer Monzón, FEDER/Gobierno de Aragón (Group B29_17R), and Ministerio de Ciencia, Innovación e Universidades (MCNU), Agencia Estatal de Investigación (SAF2017‐83120‐C2‐1‐R and PID2020-113963RB-I00). Predoctoral grants/contracts from Gobierno de Aragon (IU-M and JP) are supported by ARAID Foundation. EG is funded by Ministerio de Ciencia, Innovación y Universidades (MCNU), and Agencia Estatal de Investigación (PID2020-113963RB-I00). MA and LS are funded by Postdoctoral Juan de la Cierva Contract. SR, LC, SH, and IU-M are funded by predoctoral contracts from Aragon Government. PL is funded by FPU predoctoral grants from Ministerio de Ciencia, Innovación e Universidades. Work at the Catalan Institute of Oncology is funded by the entity, the Instituto de Salud Carlos III and Ministerio de Economia y Competitividad, and co-funded by FEDER funds—a way to build Europe (PI20/00767), CIBERESP (grant CB07/02/2005), H2020 grant MoTriColor, and the Agency for Management of University and Research Grants (AGAUR) of the Catalan Government grant 2017SGR723. This work is supported by COST Action CA17118.

## Conflict of Interest

JP reported research funding from BMS and Gilead and speaker honoraria from Gilead and Pfizer. EG reported research funding from BMS and Gilead.The funders were not involved in the study design, collection, analysis, interpretation of data, the writing of this article or the decision to submit it for publication.

The remaining authors declare that the research was conducted in the absence of any commercial or financial relationships that could be construed as a potential conflict of interest. 

## Publisher’s Note

All claims expressed in this article are solely those of the authors and do not necessarily represent those of their affiliated organizations, or those of the publisher, the editors and the reviewers. Any product that may be evaluated in this article, or claim that may be made by its manufacturer, is not guaranteed or endorsed by the publisher.
