# Supplementary material for: Adoptive NK Cell Transfer as a Treatment in Colorectal Cancer Patients: Analyses of Tumour Cell Determinants Correlating With Efficacy In Vitro and In Vivo
Source: Front Immunol. 2022 Jun 7;13:890836. doi: 10.3389/fimmu.2022.890836 (PMC9210952; doi:10.3389/fimmu.2022.890836)
Supplement: Supplementary file 1 [file DataSheet_1.pdf]

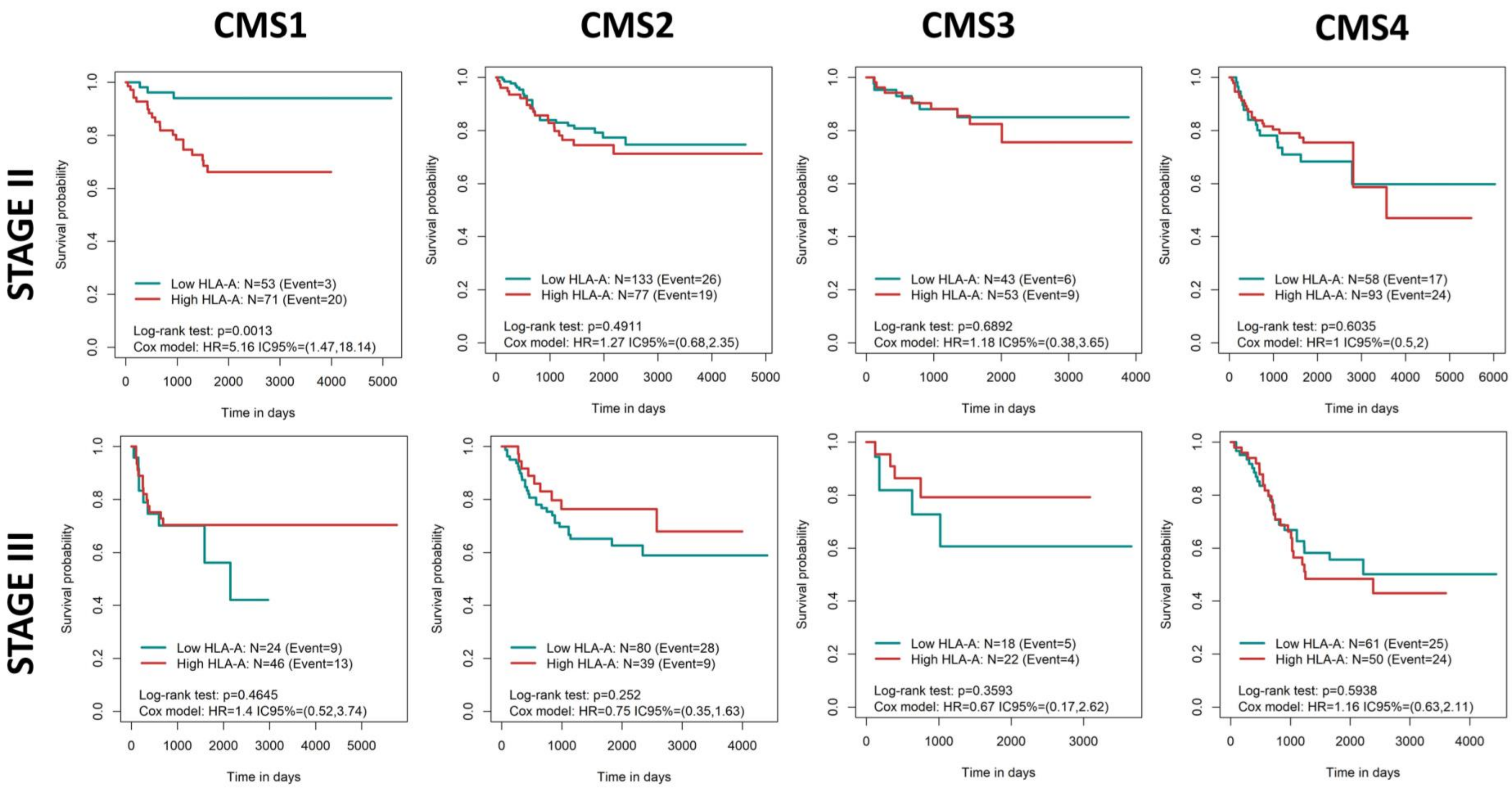

Supplementary Figure 1. Kaplan–Meier curves dividing into high and low HLA-A categories in tumors, stratifying by stage and CMS. To define low (green line) and high (red line) HLA-A categories, the median gene expression was used as cutoff, in each dataset. P-values were calculated using the binary logrank test and by fitting a Cox proportional hazards regression model stratifying by study and adjusted for age, sex and stroma.
