# Supplementary material for: Adoptive NK Cell Transfer as a Treatment in Colorectal Cancer Patients: Analyses of Tumour Cell Determinants Correlating With Efficacy In Vitro and In Vivo
Source: Front Immunol. 2022 Jun 7;13:890836. doi: 10.3389/fimmu.2022.890836 (PMC9210952; doi:10.3389/fimmu.2022.890836)
Supplement: Supplementary file 4 [file DataSheet_4.pdf]

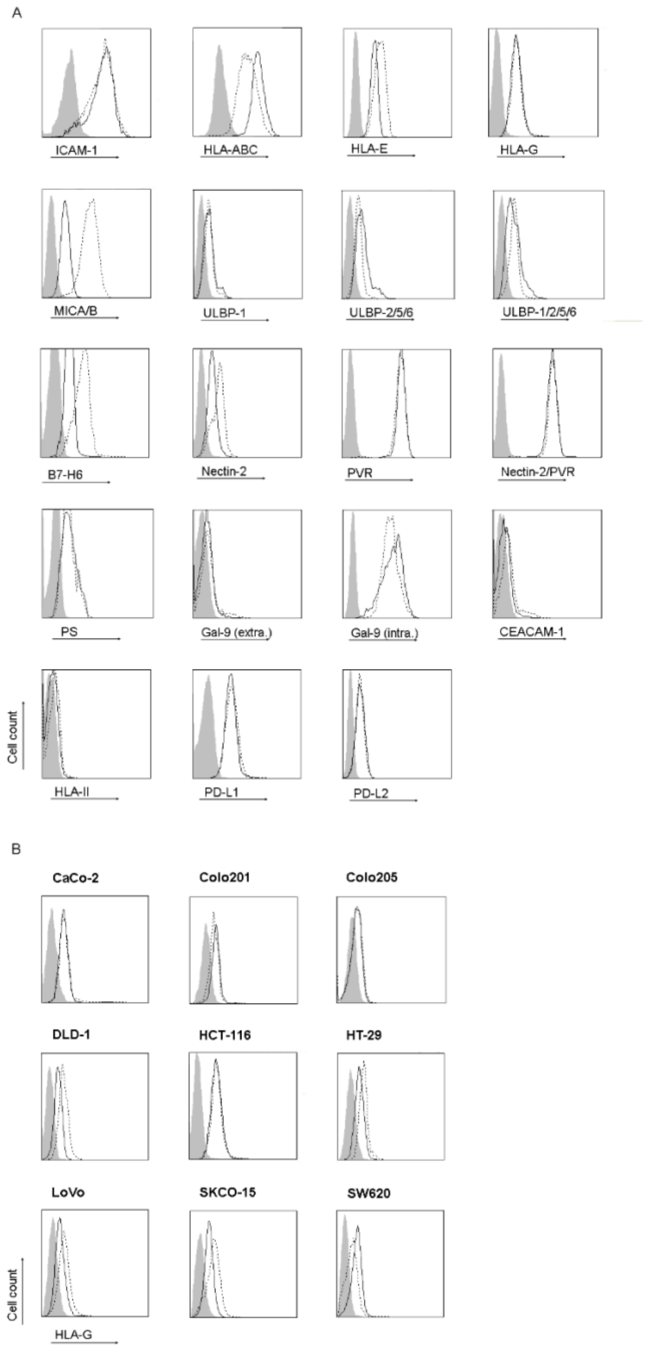

Supplementary Figure 4. Ligands for NK cell receptors expressed by CRC cell lines.
A) Histograms representing flow cytometry staining for NK cell receptors in the representative HCT-116 CRC cell line in both 2D (solid line) and 3D (dotted line) conditions. The grey histograms represent the unstained control.
B) Histograms representing HLA-G flow cytometry staining in the panel of CRC cell lines in both 2D (solid line) and 3D (dotted line) conditions. The grey histograms represent the unstained control for each cell line.
