# Supplementary material for: Adoptive NK Cell Transfer as a Treatment in Colorectal Cancer Patients: Analyses of Tumour Cell Determinants Correlating With Efficacy In Vitro and In Vivo
Source: Front Immunol. 2022 Jun 7;13:890836. doi: 10.3389/fimmu.2022.890836 (PMC9210952; doi:10.3389/fimmu.2022.890836)
Supplement: Supplementary file 5 [file DataSheet_5.pdf]

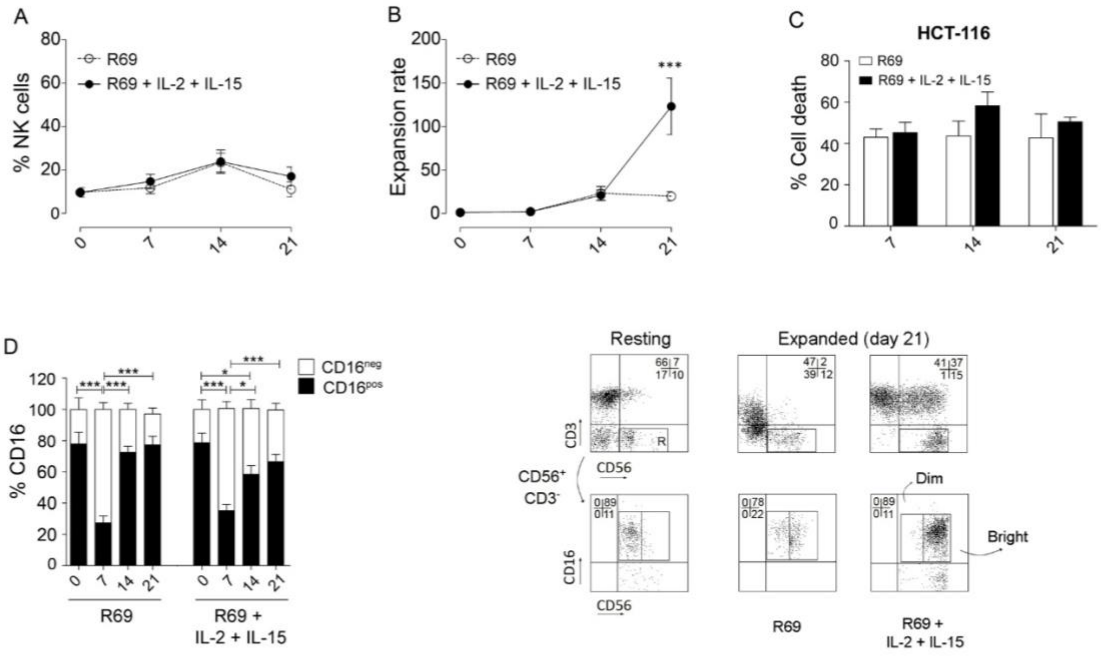

Supplementary Figure 5. Characterization of NK cell expansion protocols. PBMCs derived from HD were cultured for 21 days with LCL-EBV+ R69 feeder cells alone (white) or in combination with IL-2 (100 UI/mL) and IL-15 (5ng/mL) (black).

A) Percentage of NK cells in culture analysed by flow cytometry at different expansion timepoints. Data are presented as mean ± SEM from 8 donors in 4 independent experiments.

B) NK cell expansion rate was calculated by direct measure of PBMC expansion rate and the percentage of NK cells in culture. Data are presented as mean ± SEM from 8 donors in 4 independent experiments.

C) Cytotoxicity of activated NK cells against the CRC cell line HCT-116, determined in a 4 h assay, at a 3:1 (e:t) ratio, and at different expansion time points. Cell death was analysed by flow cytometry using Annexin-V/PS staining in the e-Fluor670 negative population. Data are presented as mean ± SEM from 4 donors in 2 independent experiments after subtraction of respective controls without effector NK cells. Cell viability without NK cells was always >85%.

D) Evolution of CD16 expression on expanded NK cells at different time points, with the histogram representing CD16 positive (black) and CD16 negative (white) populations. Dot plots indicating gating strategy are included. LCL-EBV+ R69 feeder cells alone (white) or in combination with IL-2 and IL-15 (black). Data are presented as mean ± SEM from 8 donors in 4 independent experiments.

Statistical analyses were performed by one-way or two-way ANOVA test with Bonferroni's post-test. *$p < 0.05$; **$p < 0.01$; ***$p < 0.001$.
