# Supplementary material for: Adoptive NK Cell Transfer as a Treatment in Colorectal Cancer Patients: Analyses of Tumour Cell Determinants Correlating With Efficacy In Vitro and In Vivo
Source: Front Immunol. 2022 Jun 7;13:890836. doi: 10.3389/fimmu.2022.890836 (PMC9210952; doi:10.3389/fimmu.2022.890836)
Supplement: Supplementary file 6 [file DataSheet_6.pdf]

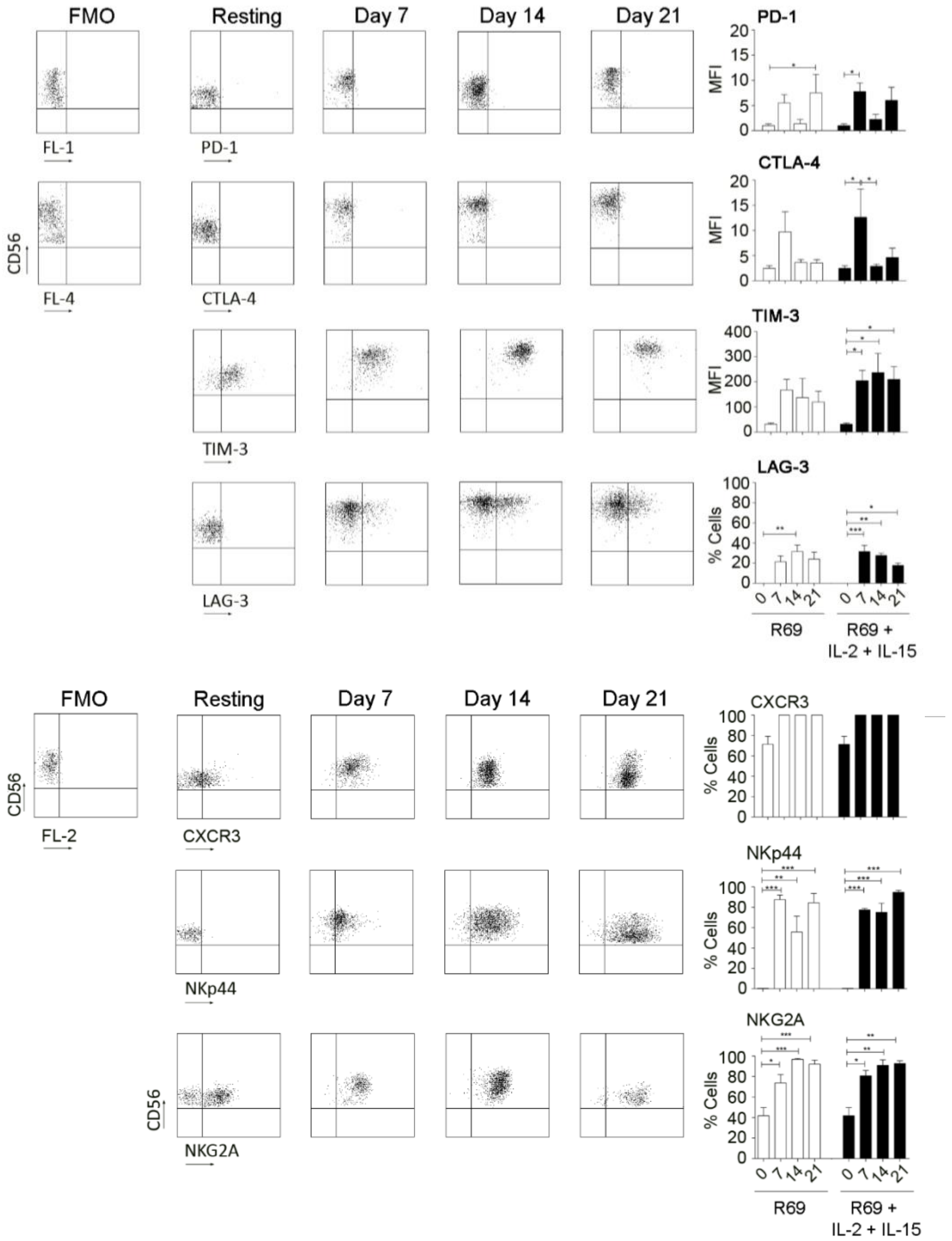

Supplementary Figure 6. Evolution of NK cell receptors in the course of NK cell expansion. PBMCs derived from HD were cultured for 21 days with LCL-EBV+ R69 feeder cells alone (white) or in combination with IL-2 (100 UI/mL) and IL-15 (5 ng/mL) (black). Expression of various conventional receptors and emerging checkpoints was analysed by flow cytometry. Representative dot plots show the expression of these receptors on the NK cell population. Histograms represent mean ± SEM from 4 donors in 2 independent experiments. Statistical analyses were performed by one-way ANOVA test with Bonferroni's post-test. *p < 0.05; **p < 0.01; ***p < 0.001.
