# Supplementary material for: Adoptive NK Cell Transfer as a Treatment in Colorectal Cancer Patients: Analyses of Tumour Cell Determinants Correlating With Efficacy In Vitro and In Vivo
Source: Front Immunol. 2022 Jun 7;13:890836. doi: 10.3389/fimmu.2022.890836 (PMC9210952; doi:10.3389/fimmu.2022.890836)
Supplement: Supplementary file 7 [file DataSheet_7.pdf]

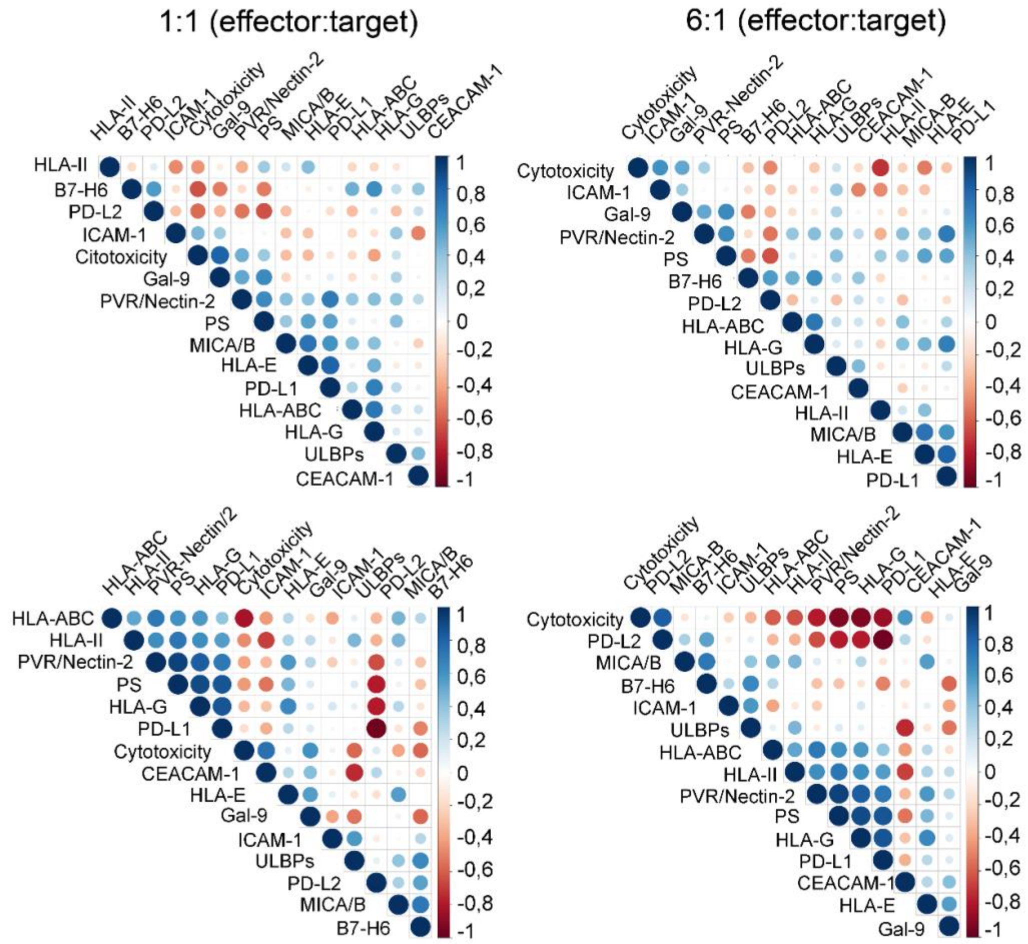

Supplementary Figure 7. Correlation between NK cell ligand expression and activated NK cell cytotoxicity. Ligand expression level and percentage of cell death at 1:1 and 6:1 (e:t) ratios were analysed in 2D and 3D models. The matrices represent positive correlation (blue) or negative correlation (red). Colour intensity and dot size show greater correlation.
