# Supplementary material for: Adoptive NK Cell Transfer as a Treatment in Colorectal Cancer Patients: Analyses of Tumour Cell Determinants Correlating With Efficacy In Vitro and In Vivo
Source: Front Immunol. 2022 Jun 7;13:890836. doi: 10.3389/fimmu.2022.890836 (PMC9210952; doi:10.3389/fimmu.2022.890836)
Supplement: Supplementary file 8 [file DataSheet_8.pdf]

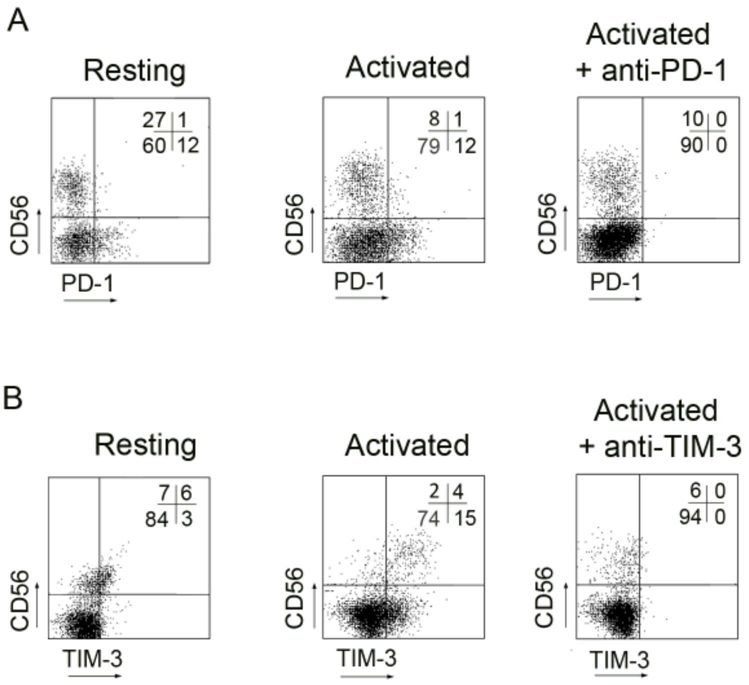

Supplementary Figure 8. Blocking ability of the antiPD-1 (Pembrolizumab) and antiTIM-3 antibodies

Determination of PD-1 (A) and TIM-3 (B) expression in resting and activated PBMCs. A fraction of activated PBMCs was incubated with either 20 µg/mL Pembrolizumab or anti-TIM3 before the staining.
