# Supplementary material for: Adoptive NK Cell Transfer as a Treatment in Colorectal Cancer Patients: Analyses of Tumour Cell Determinants Correlating With Efficacy In Vitro and In Vivo
Source: Front Immunol. 2022 Jun 7;13:890836. doi: 10.3389/fimmu.2022.890836 (PMC9210952; doi:10.3389/fimmu.2022.890836)
Supplement: Supplementary file 9 [file Table_1.docx]

**Supplementary Table 1. Clinico-pathologic features of patients in transcriptomics datasets**

| **Variable** |  | **Total** | **CLX** | **GSE13294** | **GSE14333** | **GSE17536** | **GSE39582** | **TCGA** | **P-value** |
| --- | --- | --- | --- | --- | --- | --- | --- | --- | --- |
| N |  | 1079 | 98 | 121 | 185 | 111 | 421 | 143 |  |
| Sex | Female | 490 (45.4%) | 27 (27.6%) | 60 (49.6%) | 87 (47%) | 58 (52.3%) | 188 (44.7%) | 70 (49%) | 0.0054 |
|  | Male | 589 (54.6%) | 71 (72.4%) | 61 (50.4%) | 98 (53%) | 53 (47.7%) | 233 (55.3%) | 73 (51%) |  |
| Age |  | 68 (59, 76) | 71 (65, 78) | 70 (61, 79) | 67 (58, 75) | 67 (56, 74) | 69 (59, 76) | 65 (57, 73) | 0.0015 |
| Stage | II | 684 (63.4%) | 98 (100%) | 121 (100%) | 94 (50.8%) | 55 (49.5%) | 233 (55.3%) | 83 (58%) | <0,0001 |
|  | III | 395 (36.6%) | 0 (0%) | 0 (0%) | 91 (49.2%) | 56 (50.5%) | 188 (44.7%) | 60 (42%) |  |
| Event | No | 793 (73.5%) | 76 (77.6%) | 96 (79.3%) | 137 (74.1%) | 80 (72.1%) | 285 (67.7%) | 119 (83.2%) | 0.004 |
|  | Yes | 286 (26.5%) | 22 (22.4%) | 25 (20.7%) | 48 (25.9%) | 31 (27.9%) | 136 (32.3%) | 24 (16.8%) |  |
| Time Survival |  | 1411 (720, 2010) | 1894 (1252, 2293) | 1825 (1825, 1825) | 1108 (631, 1734) | 1102 (686, 1776) | 1590 (750, 2430) | 811 (501, 1172) | <0,0001 |
| MSI | MSI | 134 (24.2%) | 0 (0%) | 64 (52.9%) | 0 (NaN%) | 0 (NaN%) | 70 (21%) | 0 (NaN%) | <0,0001 |
|  | MSS | 419 (75.8%) | 98 (100%) | 57 (47.1%) | 0 (NaN%) | 0 (NaN%) | 264 (79%) | 0 (NaN%) |  |
| MSI Imputed | MSI | 239 (22.2%) | 0 (0%) | 64 (52.9%) | 56 (30.3%) | 24 (21.6%) | 78 (18.5%) | 17 (11.9%) | <0,0001 |
|  | MSS | 840 (77.8%) | 98 (100%) | 57 (47.1%) | 129 (69.7%) | 87 (78.4%) | 343 (81.5%) | 126 (88.1%) |  |
| CMS | CMS1 | 194 (21.1%) | 6 (6.9%) | 26 (28.3%) | 34 (23.8%) | 32 (32.3%) | 72 (19.9%) | 24 (17.4%) | 3e-04 |
|  | CMS2 | 329 (35.7%) | 31 (35.6%) | 23 (25%) | 39 (27.3%) | 37 (37.4%) | 150 (41.4%) | 49 (35.5%) |  |
|  | CMS3 | 136 (14.8%) | 21 (24.1%) | 16 (17.4%) | 24 (16.8%) | 9 (9.1%) | 45 (12.4%) | 21 (15.2%) |  |
|  | CMS4 | 262 (28.4%) | 29 (33.3%) | 27 (29.3%) | 46 (32.2%) | 21 (21.2%) | 95 (26.2%) | 44 (31.9%) |  |
| Strome |  | 976 (-367, 2308) | -30 (-640, 1513) | 1438 (222, 2278) | 2545 (1680, 3255) | 1465 (744, 2405) | 794 (-216, 1894) | -1366 (-2080, -693) | <0,0001 |
| HLA-A expression |  | 12.7 (12.3, 13.2) | 10.5 (10.1, 10.8) | 12.4 (12, 12.5) | 12.5 (12.3, 12.6) | 13.3 (13.1, 13.5) | 12.9 (12.6, 13.1) | 14.8 (14.3, 15.3) | <0,0001 |
| HlLA-A expression categorical | Low | 537 (49.8%) | 49 (50%) | 60 (49.6%) | 92 (49.7%) | 55 (49.5%) | 210 (49.9%) | 71 (49.7%) | 1 |
|  | High | 542 (50.2%) | 49 (50%) | 61 (50.4%) | 93 (50.3%) | 56 (50.5%) | 211 (50.1%) | 72 (50.3%) |  |
