# Supplementary material for: Adoptive NK Cell Transfer as a Treatment in Colorectal Cancer Patients: Analyses of Tumour Cell Determinants Correlating With Efficacy In Vitro and In Vivo
Source: Front Immunol. 2022 Jun 7;13:890836. doi: 10.3389/fimmu.2022.890836 (PMC9210952; doi:10.3389/fimmu.2022.890836)
Supplement: Supplementary file 10 [file Table_2.docx]

**Supplementary Table 2. Cell culture conditions**

|  |  |  | 3D conditions | |
| --- | --- | --- | --- | --- |
| Cell line | **Origin** | **Basal medium** | **Methocell percentage** | **Incubation time (h)** |
| CaCo-2 | CRC | DMEM (Sigma)^1^ | 20% | 48 |
| Colo-201 | CRC | RPMI-1640 (Gibco) | 20% | 48 |
| Colo-205 | CRC | RPMI-1640 (Gibco) | 20% | 48 |
| DLD-1 | CRC | DMEM (Sigma) ^1^ | 20% | 48 |
| HCT-116 | CRC | DMEM (Sigma) ^1^ | 20% | 48 |
| HT-29 | CRC | DMEM (Sigma) ^1^ | 20% | 48 |
| LoVo | CRC | RPMI-1640 (Gibco) | 30% | 72 |
| SKCO-15 | CRC | DMEM (Sigma) ^1^ | 30% | 72 |
| SW620 | CRC | Leibovitz’s (Gibco) | 30% | 72 |
| R69 | Lymphoblastoid | RPMI-1640 (Gibco) | - | - |

^1^ DMEM high glucose (4500 mg/L)
