# Supplementary material for: Adoptive NK Cell Transfer as a Treatment in Colorectal Cancer Patients: Analyses of Tumour Cell Determinants Correlating With Efficacy In Vitro and In Vivo
Source: Front Immunol. 2022 Jun 7;13:890836. doi: 10.3389/fimmu.2022.890836 (PMC9210952; doi:10.3389/fimmu.2022.890836)
Supplement: Supplementary file 11 [file Table_3.docx]

**Supplementary Table 3. Mutational status of the CRC cell lines collection**

| Cell line | Mismatch repair | Hyper-methylation | KRAS | BRAF | PIK3 | PTEN | P53 |
| --- | --- | --- | --- | --- | --- | --- | --- |
| CaCo-2 | MSS | CIMP - | *wt* | *wt* | *wt* | *wt* | E204X |
| CoLo-201 | MSS | CIMP + | *wt* | V600E | *wt* | *wt* | Y103F or  Y103pf*37 |
| CoLo-205 | MSS | CIMP + | *wt* | V600E | *wt* | *wt* | Y103F or  Y103pf*37 |
| DLD-1 | MSI | CIMP + | G13D | *wt* | E545K  D549N | *wt* | S241F |
| HCT-116 | MSI | CIMP + | G13D | *wt* | H1047R | *wt* | *wt* |
| HT-29 | MSS | CIMP + | *wt* | V600E | P449T | *wt* | R273H |
| LoVo | MSI | CIMP - | G13D  A14V | *wt* | *wt* | *wt* | *wt* |
| SKCO- 15 | *-* | *-* | *-* | *-* | *-* | *-* | *-* |
| SW620 | MSS | CIMP - | G12V | *wt* | *wt* | *wt* | R273H  P309S |

**CIMP:** dCpG Island Methylator Phenotype; **MSI:** Microsatellite Instability; **MSS:** Microsatellite Stable
