# Supplementary material for: Adoptive NK Cell Transfer as a Treatment in Colorectal Cancer Patients: Analyses of Tumour Cell Determinants Correlating With Efficacy In Vitro and In Vivo
Source: Front Immunol. 2022 Jun 7;13:890836. doi: 10.3389/fimmu.2022.890836 (PMC9210952; doi:10.3389/fimmu.2022.890836)
Supplement: Supplementary file 3 [file DataSheet_3.pdf]

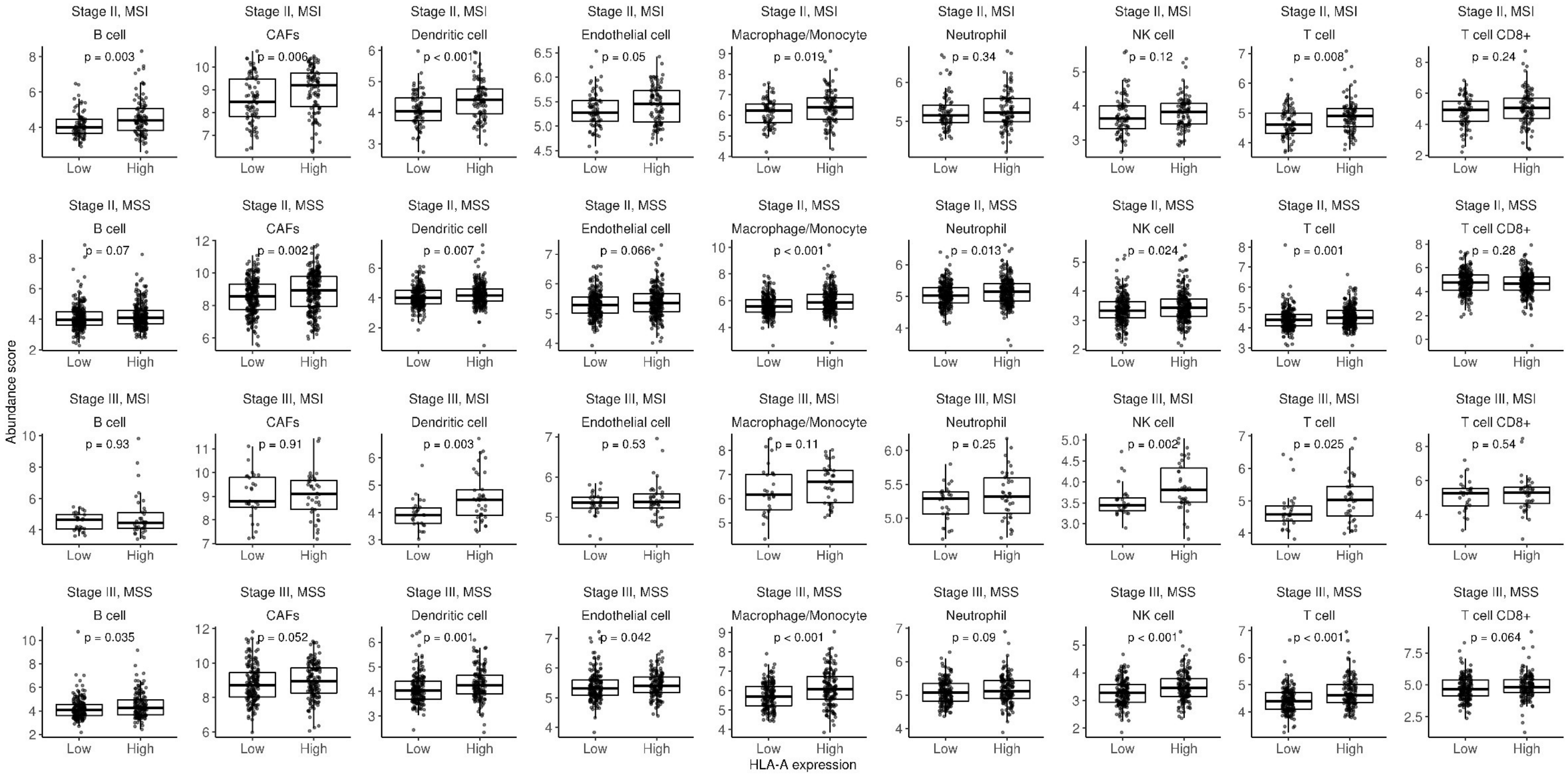

Supplementary Figure 3. Box plots showing enrichment scores of 18 different stromal and immune cell-types across CRC patients stratified by stage and MSS/MSI status. Enrichment scores were calculated applying GSVA method over gene sets of immune infiltrate markers extracted from Consensus TME tool. Differences were assessed using non-parametric Kruskal-Wallis and Tukey's tests (* $p \leq 0.05$; ** $p \leq 0.01$; *** $p \leq 0.001$; **** $p \leq 0.\ 0001$
